# Force‐Induced Selective Carbon‐Carbon Bond Cleavage in Mechanoresponsive Topochemical Polymers

**DOI:** 10.1002/adma.202510482

**Published:** 2025-09-18

**Authors:** Zitang Wei, Hanul Kim, Nazmul Haque, Qixuan Hu, Ke Ma, Kang Wang, Shuchen Zhang, Xuyi Luo, Yoon Ho Lee, Siyoung Q. Choi, Chelsea S Davis, Brett M. Savoie, Letian Dou

**Affiliations:** ^1^ Davidson School of Chemical Engineering Purdue University West Lafayette IN 47907 USA; ^2^ Department of Chemical Engineering Massachusetts Institute of Technology Cambridge MA 02139 USA; ^3^ Department of Chemical and Biomolecular Engineering Korea Advanced Institute of Science and Technology (KAIST) Daejeon 34141 Republic of Korea; ^4^ School of Materials Engineering Purdue University West Lafayette IN 47907 USA; ^5^ Global Institute of Future Technology Shanghai Jiao Tong University Shanghai 200240 China; ^6^ Key Laboratory of Photochemistry Institute of Chemistry Chinese Academy of Sciences Beijing 100190 China; ^7^ State Key Laboratory of Precision and Intelligent Chemistry Department of Materials Science and Engineering School of Chemistry and Materials Science University of Science and Technology of China Hefei 230026 China; ^8^ Department of Chemistry Stanford University Stanford CA 94305 USA; ^9^ Department of Materials Science & Engineering Sungshin Women's University Seoul 01133 Republic of Korea; ^10^ Department of Mechanical Engineering University of Delaware Newark DE 19716 USA; ^11^ Department of Chemical and Biomolecular Engineering University of Notre Dame Notre Dame IN 46556 USA; ^12^ Department of Chemistry Purdue University West Lafayette IN 47907 USA; ^13^ Department of Chemistry Emory University Atlanta GA 30322 USA

**Keywords:** composite films, mechanoresponsive materials, stress visualization, topochemical polymers

## Abstract

Mechanoresponsive polymeric materials that respond to mechanical deformation are highly valued for their potential in sensors, degradation studies, and optoelectronics. However, direct visualization and detection of these responses remain significant obstacles. In this study, novel mechanoresponsive polybiidenedionediyl (PBIT) derivative topochemical polymers are developed that depolymerize under mechanical forces, exhibiting a distinct and irreversible color change in response to grinding, milling, and compression. This color change is attributed to the alteration of polymer backbone conjugation during elongated Carbon‐Carbon (C─C) single bond cleavage. Quantum chemical pulling simulations on PBIT polymers reveals a force range of 4.3–5.0 nN associated with the selective cleavage of elongated C─C single bonds. This force range is comparable to that observed for typical homolytic mechanophores, supporting the mechanistic interpretation of homolytic bond scission under mechanical stress. C─C bond cleavage kinetic studies of PBIT under compression indicates that strong interchain interactions significantly increase the pressure needed to cleave the elongated C─C bonds. Additionally, PBIT polymer thin films are composited with polydimethylsiloxane to create free‐standing and robust thin films, which can serve as ink‐free and rewritable paper for writing and stress visualization applications. This advancement opens new possibilities for utilizing crystalline and brittle topochemical polymers in practical applications.

## Graphical abstract

A new class of mechanoresponsive polymers based on polybiidenedionediyl (PBIT) derivatives exhibits distinct color changes upon mechanical stimuli such as grinding, milling, and compression. The response arises from depolymerization and backbone conjugation changes. A PBIT–PDMS composite film enables ink‐free, rewritable paper, offering a simple and direct platform for stress visualization.

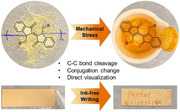

## 1 Introduction

Mechanoresponsive polymers represent a significant class of materials capable of altering their properties in response to predefined mechanical stimuli in a highly selective and predictable manner.^[^ ^1^, ^2^, ^3^, ^4^, ^5^, ^6^ ^]^ Typical responses, such as changes in chemical reactivity,^[^ ^7^, ^8^, ^9^ ^]^ optical properties,^[^ ^10^, ^11^, ^12^, ^13^ ^]^ electrical conductivity,^[^ ^14^, ^15^ ^]^ magnetic response,^[^ ^16^, ^17^ ^]^ and other characteristics under mechanical stimuli, have been extensively studied. These response characteristics have been utilized to understand polymer mechanics,^[^ ^18^, ^19^, ^20^ ^]^ direct polymer degradation and reconstruction,^[^ ^21^, ^22^, ^23^, ^24^ ^]^ and develop force sensors.^[^ ^25^, ^26^, ^27^, ^28^ ^]^ Despite substantial advancements, effectively leveraging this knowledge for the rational design of functional mechanoresponsive materials remains a considerable challenge. Furthermore, translating the remarkable properties of mechanoresponsive polymers into practical real‐world applications continues to be a challenging endeavor.

Colorimetric and luminescent changes are frequently observed as a result of microscopic structural transitions triggered by external mechanical stimuli. These mechanochromic responses serve as visual indicators of mechanical stress. For instance, shear forces, such as rubbing or grinding, are typically detected through changes in luminescence color,^[^ ^29^, ^30^, ^31^, ^32^ ^]^ whereas compressive stress is often manifested via aggregation‐induced emission.^[^ ^33^, ^34^ ^]^ To harness these effects, mechanoresponsive polymers are commonly engineered by incorporating mechanophores into polymer chains, crosslinked networks, or interfacial regions.^[^ ^1^ ^]^ Among the various design strategies, exotic mechanophores have been embedded into well‐established polymer matrices, including poly(methyl acrylate) (PMA),^[^ ^10^ ^]^ poly(methyl methacrylate) (PMMA),^[^ ^35^ ^]^ polystyrene (PS),^[^ ^36^ ^]^ polydimethylsiloxane (PDMS),^[^ ^37^ ^]^ and epoxy resins.^[^ ^38^ ^]^ These systems often require sophisticated molecular architectures and catalytic conditions to achieve effective mechanical responsiveness. Additionally, when chromophores are integrated into elastic polymers, applied tensile stress can be visualized through changes in visible and luminescent colors—although external light sources are frequently necessary to observe these effects.

In this study, we present a novel class of mechanoresponsive polymeric materials based on polybiidenedionediyl (PBIT) derivative, which exhibit immediate and distinct color changes in response to mechanical stimuli. Unlike conventional systems, these PBIT polymers do not require chemical integration into existing polymer matrices. Instead, they respond directly to mechanical forces—such as grinding, ball milling, or hard pressing—via the cleavage of elongated carbon–carbon (C─C) single bonds linking monomers. Constrained Geometry simulate External Force (CoGEF) simulations performed on PBIT single crystals indicate that a force range of 4.3–5.0 nN is sufficient to selectively cleave these elongated C─C single bonds, comparable to the force required for typical homolytic mechanophores. Kinetic analyses of C─C bond cleavage across various PBIT materials reveal that both shorter C─C bonds connecting monomer units and strong interchain interactions among side chains can elevate the mechanical force threshold required for bond scission and subsequent depolymerization. Furthermore, we fabricated a PBIT‐PDMS composite thin film through ultrasonication and blade coating techniques. These composite films function as ink‐free, rewritable paper for writing and stress visualization applications. This work introduces a new class of mechanoresponsive polymers characterized by rapid and unambiguous colorimetric responses, offering a promising platform for the practical deployment of otherwise challenging topochemical polymer single crystals.

## 2 Results and Discussion

First reported in 2014, polybiidenedionediyl (PBIT) derivatives represent a remarkable class of polymers capable of undergoing quantitative topochemical polymerizations triggered by visible light.^[^ ^39^ ^]^ Through a simple solvent evaporation process, high‐quality orange monomer crystals exceeding 5 mm in length were obtained. These crystals, upon illumination with visible light, were quantitatively converted into light yellow polymer crystals (Figure S1, Supporting Information). Analysis of PBIT single crystal structures revealed that the monomer backbones are connected by elongated Carbon‐Carbon (C─C) single bonds with a bond length of ≈1.60 Å (Figures S2–S4, Supporting Information). Notably, these elongated C─C bonds in PBIT can be selectively cleaved under heating conditions in organic solvents, resulting in near‐quantitative depolymerization yields.^[^ ^40^, ^41^ ^]^ However, a significant drawback of the previous demonstration of elongated C─C bond cleavage and depolymerization in PBIT is the requirement for high boiling point solvents, such as anisole or dichlorobenzene, for selective C─C bond cleavage. The necessity of heating and organic solvents renders the polymer depolymerization process costly. And the fact that PBIT single crystals are not soluble in any common organic and inorganic solvents makes it challenging to utilize PBIT polymers in real‐world applications.^[^ ^39^, ^40^, ^42^ ^]^

**Figure 1:**
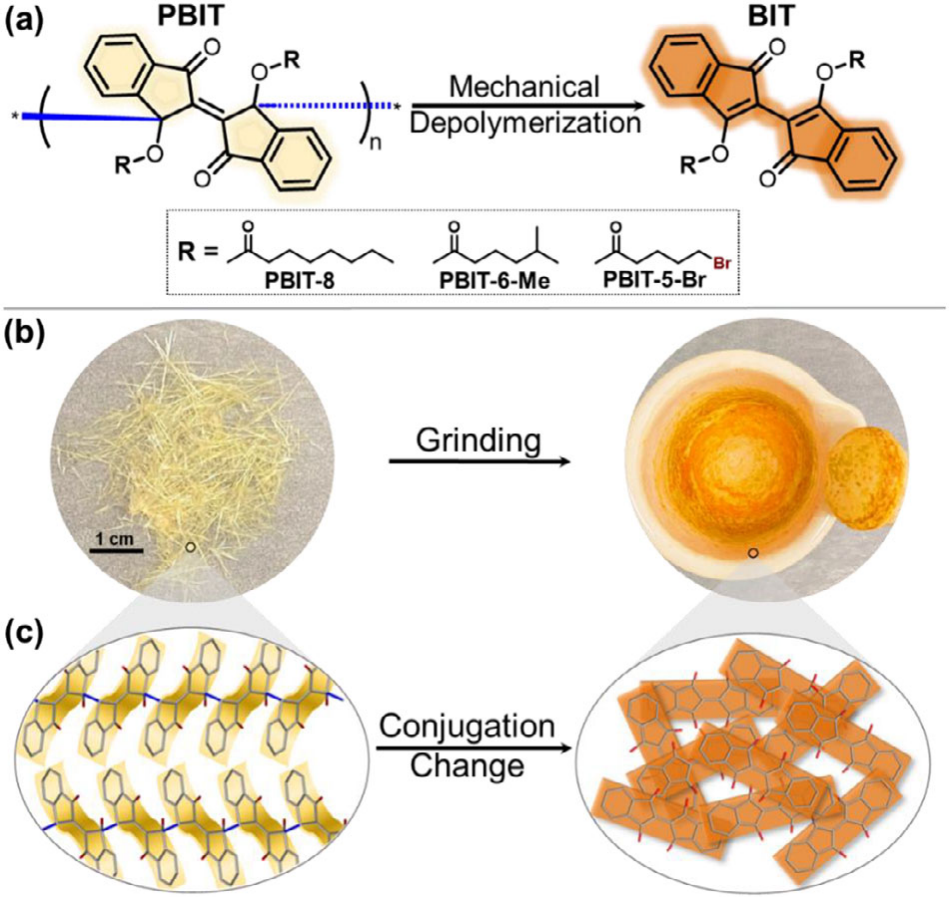
Chemical Structures and Bulk Polymer Samples of PBIT. a) Chemical structure of the PBIT polymer and its mechanically depolymerized BIT monomer, with different R groups highlighted in the dotted box. b) Photographs depicting the PBIT polymer before (left) and after grinding (right). c) Single crystal structures of PBIT polymer with a non‐flat, broken conjugation backbone (left) and BIT monomer with a flat, conjugated backbone (right).

Analysis of the single crystal structures of PBIT revealed that the elongated C─C bonds are tightly surrounded by multiple rings and side chains, making them susceptible to breaking via twisting or stretching of the crystal lattices (Figures S2–S4, Supporting Information). We discovered that elongated C─C single bonds in PBIT crystals can be mechanically cleaved in the solid state. Using a simple pestle and mortar for hand grinding, PBIT crystals immediately changed color from light yellow to bright orange (**Figure** **1**). The ^1^H nuclear magnetic resonance (NMR) spectrum of the orange powders post‐grinding was identical to that of fresh biidenedionediyl (BIT) derivative monomers (Figures S5–S7, Supporting Information), confirming the formation of monomers after grinding. By performing multiple grindings followed by rinsing the residue with acetone, the mechanical depolymerization yield exceeded 60% (details on the multiple grindings and depolymerization yield process can be found in Supporting Information and Figure S8, Supporting Information). In addition to hand grinding, ball milling and compression of PBIT polymers were also tested, resulting in similar depolymerization behavior and color changes (Figures S9–S11, Supporting Information). These results demonstrate that elongated C─C bond cleavage of PBIT can be achieved under various conditions. To eliminate the possibility of elongated C─C bond breakage due to heat generated from mechanical stress, ball milling test of PBIT polymers was conducted while cooling the instrument to 20 °C using an industrial chiller (Figure S9, Supporting Information). After ball milling for 20 min at 20 °C, elongated C─C bond cleavage was observed, indicated by color change. This result confirms that the bonds were cleaved by mechanical stress rather than heat.

Mechanical depolymerization of PBIT polymers under varying force conditions reveals notable differences in side product formation. ^1^H NMR spectra of BIT‐8 monomers obtained via hand grinding, ball milling, and hard pressing show minor peaks ≈3.5 and 8 ppm, absent in the pristine monomer (Figure S11, Supporting Information). These peaks, though not assigned due to their low integration (<5%), are likely indicative of side reactions triggered by excessive mechanical force. Among the methods, hard pressing produced the greatest number and intensity of unknown peaks (Figure S11c, Supporting Information), suggesting a higher degree of side reactions. Ball milling (Figure S11b, Supporting Information) also showed more pronounced side peaks compared to hand grinding (Figure S11a, Supporting Information), which applies relatively limited force. The data suggest that optimizing the applied mechanical force is critical for achieving efficient depolymerization while minimizing undesired side reactions.

The pronounced color change from light yellow (PBIT polymer) to bright orange (BIT monomer) serves as a clear visual indicator of C─C bond cleavage. This transformation originates from the depolymerization process and the distinct single‐crystal structures of the PBIT polymers and BIT monomers. Structural analysis of the PBIT polymer reveals that two indane moieties along the polymer backbone are connected via a double bond, resulting in a non‐planar conformation and a light‐yellow appearance (Figure 1b,c). In contrast, the BIT monomer adopts a planar, fully conjugated structure featuring butadiene moieties, which accounts for its bright orange coloration (Figure S1, Supporting Information). During depolymerization, diradical intermediates are generated, followed by the reformation of the conjugated butadiene units.^[^ ^43^ ^]^ This reconstruction restores the extended π‐conjugation of the original monomer backbone, thereby producing the observed color change. Fourier Transform Infrared (FT‐IR) spectroscopy was performed on PBIT‐8 polymer crystals, pristine BIT‐8 monomer crystals, and recovered BIT‐8 powders obtained after mechanical depolymerization. As shown in Figure S12a (Supporting Information), the FT‐IR spectra of the pristine BIT‐8 monomer and the recycled BIT‐8 powder are nearly identical, confirming that the BIT‐8 monomer was successfully recovered following mechanical depolymerization. In addition, comparison with the FT‐IR spectrum of the PBIT‐8 polymer crystal reveals a distinct peak at 1550 cm^−1^ in the BIT‐8 monomer spectrum, corresponding to the C═C stretching vibration of the conjugated five‐membered ring on the indene backbone (Figure S12b, Supporting Information). This peak is absent in the polymer spectrum, indicating disruption of conjugation upon polymerization.

**Figure 2:**
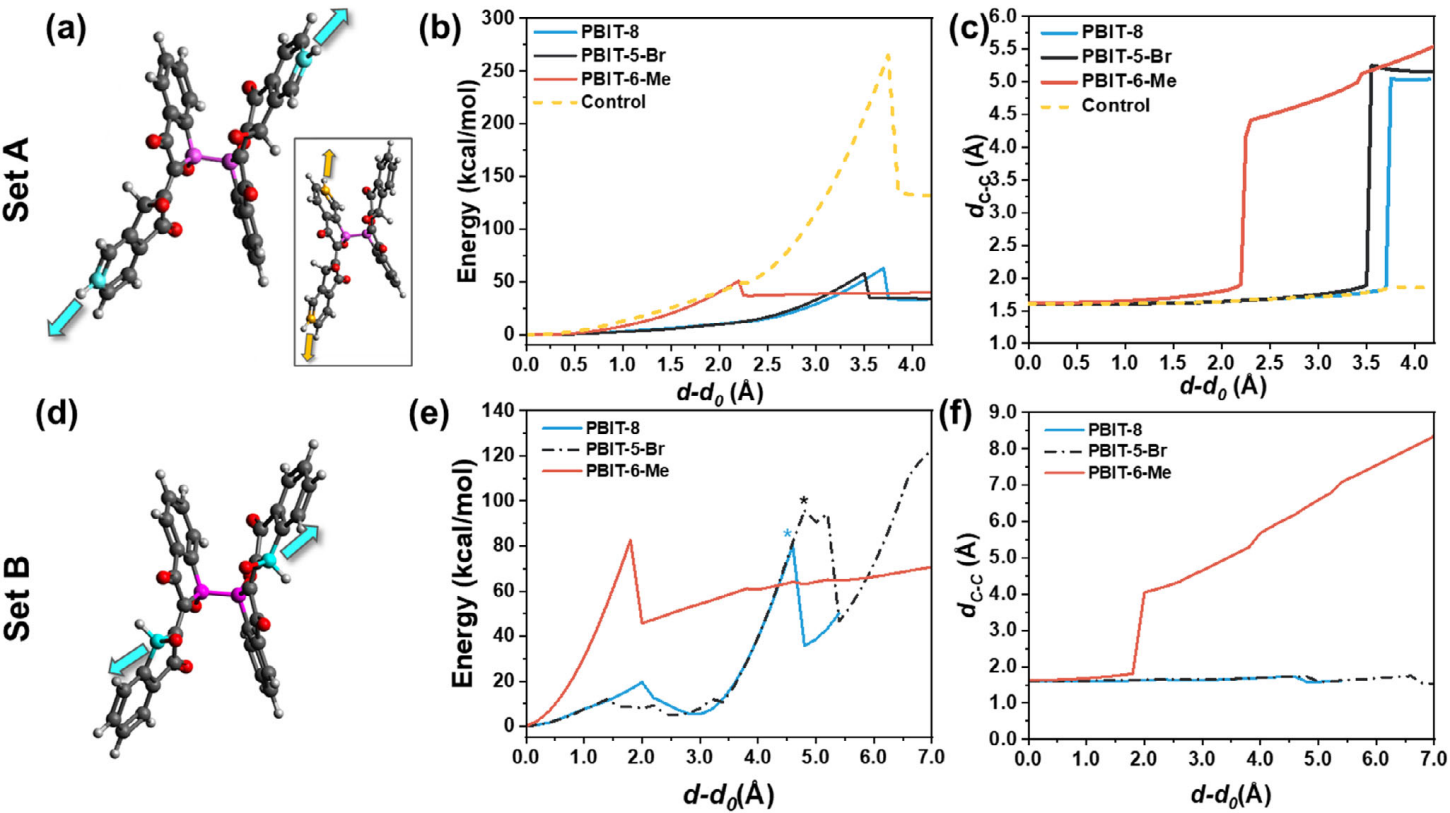
DFT simulation of mechanical depolymerization with different pulling handle atoms. a) Initial geometry of PBIT‐8, with the anchoring and central C‐C atoms colored in cyan and magenta, respectively (Set A). The inset shows the control simulation, where strain is applied within a monomer, with anchoring atoms in yellow. b) Energy and c) central C─C bond length profile of PBITs. Dashed lines present control simulations. d) Initial geometry of PBIT‐8, with the different anchoring atoms and central C‐C atoms colored in cyan and magenta, respectively (Set B). e) Energy and f) central C─C bond length profile of PBITs with different anchoring atoms. Peaks with “*” denote states where reactions different from the C─C bond scission occur. Carbon, oxygen, and hydrogen atoms are shown in black, red, and white. Alkyl side chains are omitted for clarity.

Our previous studies on PBIT polymer design indicated that altering the side chains of BIT monomers can significantly enhance the mechanical properties of the resulting polymer crystals.^[^ ^42^ ^]^ In this study, PBIT with three different side chains—linear 8‐carbon aliphatic side chain (PBIT‐8), branched 7‐carbon side chain (PBIT‐6‐Me), and bromine‐substituted side chain (PBIT‐5‐Br) were tested for mechanical grinding (**Figure** **1a**). All three polymers exhibited the same color‐change phenomenon and depolymerization after grinding (Figure S13, Supporting Information). To verify the C─C bond cleavage at the density functional theory (DFT) level and differentiate the mechanical forces required among various PBIT polymers, a Constrained Geometry Simulate External Force (CoGEF) simulation associated with D3 dispersion correction was conducted on PBIT‐8, PBIT‐6‐Me, and PBIT‐5‐Br.^[^ ^44^, ^45^ ^]^ Despite the known limitations of the CoGEF method, including its inability to account for thermal effects, tendency to overestimate rupture forces, and potential inaccuracies in specific cases,^[^ ^46^ ^]^ this static computational approach remains a valuable tool for assessing the relative mechanochemical behavior of mechanophores. As confirmed in prior studies,^[^ ^42^ ^]^ CoGEF enables qualitative comparisons of bond rupture tendencies under constrained geometries. It should be noted that the intermolecular interactions depicted in reference^[^ ^42^ ^]^ are not present in our simulations, as all calculations were performed under vacuum conditions. The optimized structure was gradually stretched to imitate the deformation of the molecular structure driven by external forces. During this procedure, the profiles of energy and C─C bond distance (d_C‐C_) were monitored. Also, to explore the different choice in anchoring atoms and stretching direction, two sets of simulations were conducted (**Figure** **2**). In set A (Figure 2a–c), strain was applied by elongating the terminal atoms while the set B (Figure 2d–f) elongates atoms which are the positions next monomer directly attached. The pulling direction of set A is off from the direction the backbone aligns and such “sliding” between repeating units is one of common mechanisms to break the polymeric crystals.^[^ ^47^, ^48^, ^49^, ^50^, ^51^, ^52^ ^]^ During the elongation, the other atoms are allowed to fully relax.

Figure 2b displays the energy profile of the set A CoGEF simulation, where a characteristic peak and plateau indicate force‐relaxation through bond breakage. Slope analysis at the peak revealed that a force of 4.3–5.0 nN was required for bond cleavage, closely matching the force (4.2 ± 0.8 nN) needed for homolytic mechanophores, which are molecules that can be mechanically broken down and have been experimentally validated.^[^ ^15^, ^31^, ^53^, ^54^, ^55^, ^56^, ^57^, ^58^, ^59^ ^]^ In Figure 2c, the d_C‐C_ exhibits significant elongation beyond 4 Å, leading to cleavage. Similar to other mechanophores, the findings confirm that elongated C─C bonds in PBIT undergo mechanical cleavage. After dissociation, homolytic cleavage was verified, yielding two identical radicals, which are checked by unrestricted energy calculation and orbital analysis. The detailed calculation results determining the number of radicals and orbital structure is provided in Figure S14 (Supporting Information). Variations among molecules are attributed to second‐order packing and side‐chain effects as the mechanophore is conserved across the series (details about DFT calculations can be found in Figures S15, S16 and Videos S1 and S2, Supporting Information).

The selective occurrence of C─C bond cleavage in PBIT has been highlighted through comparative analysis of control simulations. In PBIT‐6‐Me, elongation within the monomer, as depicted by the yellow dotted lines in Figure 2b,c, showed that bond stretching remain confined to the monomer. Cleavage required ≈11 nN, which was significantly higher than the force needed for depolymerization. This implies that elongated C─C bond cleavage proceeds the dissociation of other bonds, enabling selective depolymerization without breaking additional bonds. This observation confirms that PBIT polymers can undergo efficient depolymerization through elongated C─C bond cleavage when a mechanical force is applied. In set B, however, only PBIT‐6‐Me exhibited C─C bond cleavage, while other PBITs’ d_C‐C_ remained near 1.7 Å (Figure 2d,e). The energy peaks observed for PBIT‐8 and PBIT‐5‐Br are attributed to side reactions such as side‐chain reattachment and ring cleavage (detailed reactants and products are shown in Figure S17 and Video S3, Supporting Information). These results indicate that C─C bond cleavage is strongly influenced by the directionality of the applied mechanical force. Specifically, the off‐axis sliding geometry (Set A) significantly reduces the bond rupture threshold compared to the backbone‐aligned stretching configuration (Set B). This suggests that shear‐like forces acting between repeating units are more effective in inducing bond scission than purely tensile forces.

Another important finding from the set A is that the force required to break elongated C─C bond varies with different PBIT side chains. As shown in Figure 2b, PBIT‐6‐Me requires the lowest force (Fmax = 4.3 nN); in comparison, PBIT‐8 and PBIT‐5‐Br need 4.9 nN and 4.8 nN, respectively. Similarly, Figure 2c shows that the d_C‐C_ at C─C bond cleavage is ≈4 Å for PBIT‐6‐Me, compared to 5 Å for PBIT‐8 and PBIT‐5‐Br. Previous measurements of the elastic modulus of PBIT single crystals revealed a consistent trend, with average values of 5.9 GPa for PBIT‐6‐Me, 8.4 GPa for PBIT‐8, and 10.6 GPa for PBIT‐5‐Br. These experimental results align with the relative mechanical robustness observed across the different PBIT variants. However, it is important to note that CoGEF simulations have inherent limitations. Specifically, they do not account for dynamic or stochastic bond rupture processes and are constrained by their static nature and lack of thermal effects. As such, while CoGEF provides qualitative insights into bond rupture tendencies under constrained geometries, its predictions should be interpreted with caution. To more accurately evaluate the mechanical depolymerization behavior of PBIT polymers, CoGEF results should be complemented with additional structural analyses, including assessments of van der Waals interactions and hydrogen bonding networks.^[^ ^42^ ^]^

**Figure 3:**
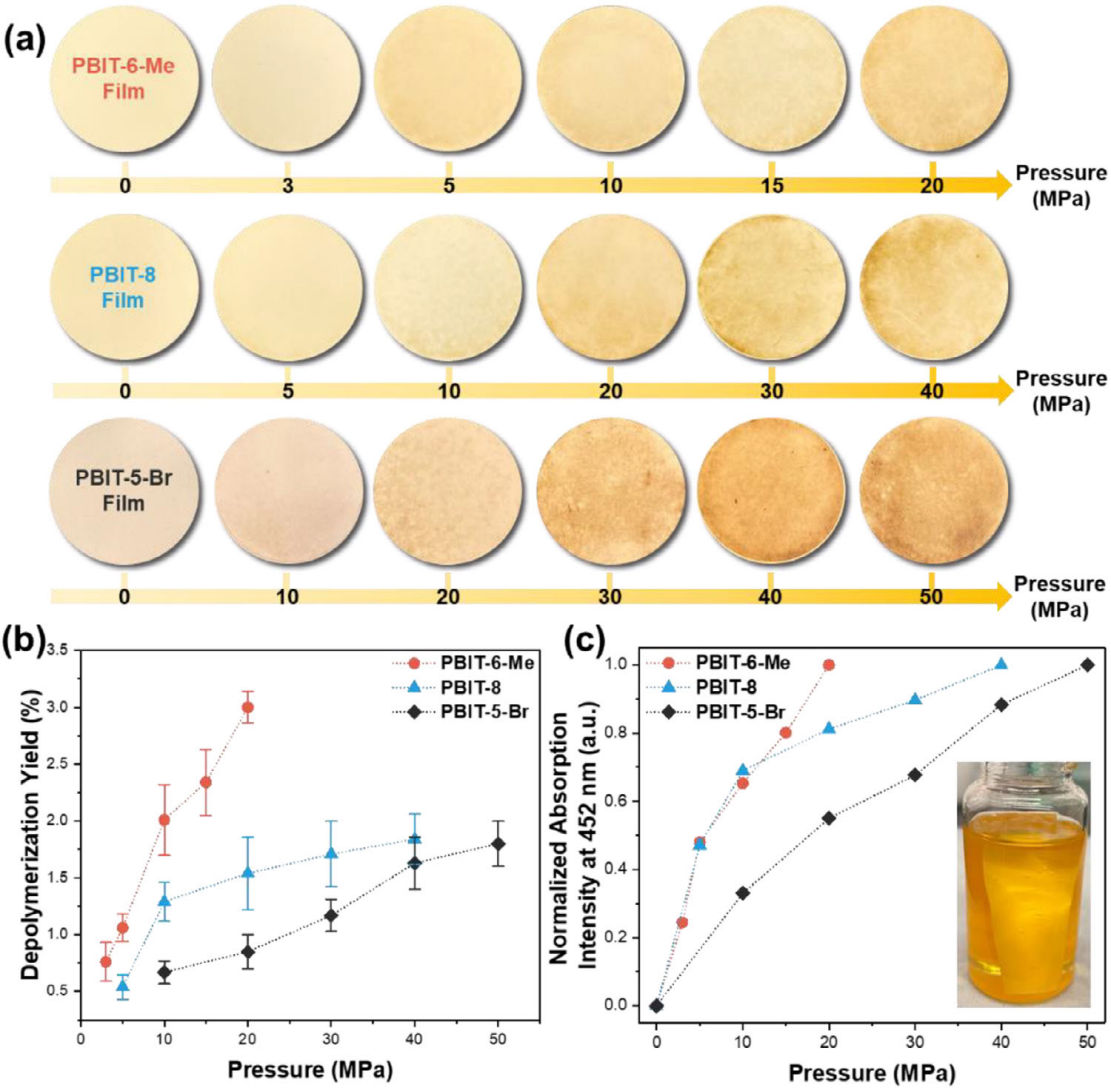
Quantitative analysis of PBIT thin films mechanical depolymerization kinetics under compression. a) Photographs of PBIT‐6‐Me, PBIT‐8, and PBIT‐5‐Br films subjected to various hard pressing pressures. All films possess a uniform diameter of 35 mm. b) Mechanical depolymerization kinetics of PBIT‐8, PBIT‐6‐Me, and PBIT‐5‐Br thin films under different pressures. c) Normalized absorption intensity at 452 nm of the resulting depolymerized monomer solutions in DCM, corresponding to the pressure applied on the three different PBIT films. The inset shows a photograph of the PBIT‐8 film after hard pressing and subsequent immersion in DCM, with the orange color indicating the dissolution of the mechanically depolymerized BIT‐8 monomer.

To experimentally quantify the force required to break elongated C─C bonds among PBIT polymers, various PBIT crystals were fabricated into free‐standing thin films via ultrasonication and vacuum filtration (details about thin film fabrication can be found in Supporting Information). Different compression forces were then applied to the films using an electromechanical universal testing machine (Figure S18, Supporting Information). During the hard pressing, the initiation pressure to cleave elongated C─C bonds in PBIT thin films was defined as the pressure at which a noticeable color change occurred on the film, corresponding to a thin film weight loss percentage of ≈0.6‐0.7% via depolymerization (details about thin film weight loss percentage calculations after hard pressing can be found in Supporting Information). As illustrated in **Figure** **3**a, the PBIT‐6‐Me film exhibited the lowest initiation pressure of only 3 MPa. In comparison, PBIT‐8 and PBIT‐5‐Br films required higher initiation pressures of 5 MPa and 10 MPa, respectively. As the force applied to the films increased, more polymer in the thin films underwent depolymerization, indicated by a more significant color change on the film surface. When the film color change reached saturation, the corresponding saturation pressures for the three different PBIT films were measured as 20 MPa (PBIT‐6‐Me films), 40 MPa (PBIT‐8 films), and 50 MPa (PBIT‐5‐Br films), respectively. Among the polymer thin films of the three different PBIT polymers, varying initiation and saturation pressures were observed. To further study the kinetics of elongated C─C bond cleavage under hard pressing, the C─C bond cleavage and depolymerization rates were examined by tracking the weight loss percentage of films after compression (Figure 3b) and the UV–vis absorption change of depolymerized monomers dissolved in solution (Figure 3c). As shown in Figure 3b, for PBIT‐6‐Me films, a depolymerization yield of 3% was achieved when the saturation pressure reached 20 MPa. In contrast, for PBIT‐8 and PBIT‐5‐Br, a depolymerization yield of only ≈1.8% was achieved at the saturation pressure. The differences in depolymerization yields among PBIT polymer films subjected to hard pressing are closely associated with variations in the elongated C─C bond lengths within the corresponding PBIT polymer crystals. Specifically, PBIT‐6‐Me exhibits the longest C─C bond length at 1.62 Å, whereas both PBIT‐8 and PBIT‐5‐Br share a shorter bond length of 1.606 Å. As previously reported,^[^ ^40^, ^42^ ^]^ longer C─C bonds generally correspond to lower bond dissociation energies, which facilitates bond cleavage under mechanical stress and leads to higher depolymerization yields. Additionally, variations in interchain interactions among PBIT polymer chains significantly influence depolymerization yield. For instance, the two freely rotating methyl groups on the side chain of PBIT‐6‐Me disrupt polymer packing, facilitating bond cleavage. In contrast, bromine atoms on the side chain of PBIT‐5‐Br promote directional C–H···Br interactions, which help stabilize the one‐dimensional stacking of polymer backbones and reduce depolymerization efficiency.^[^ ^42^ ^]^ After hard pressing, the films were shredded and carefully washed with the same amount of dichloromethane (DCM) to remove any depolymerized monomers. The UV–vis absorption spectra of the resulting depolymerized monomer solutions in DCM after washing were then measured (Figures S19–S21, Supporting Information). Figure 3c depicts the normalized absorption intensity at 452 nm of the resulting depolymerized monomer solutions in DCM with respect to the pressure applied on films for the three PBIT samples. It is evident that the rate of absorption intensity change for PBIT‐6‐Me is much faster than for the other two PBIT samples, confirming previous results that PBIT‐6‐Me requires lower forces to break the elongated C─C bonds for depolymerization. It is noteworthy that PBIT‐6‐Me and PBIT‐8 exhibit similar rates of change in absorption intensity within the initial 10 MPa range of hard pressing. This similarity can be attributed to the presence of aliphatic side chains of comparable lengths in both polymers. The relatively weak van der Waals interactions among these chains provide similar resistance to mechanical force, resulting in comparable thresholds for initiating depolymerization.

**Figure 4:**
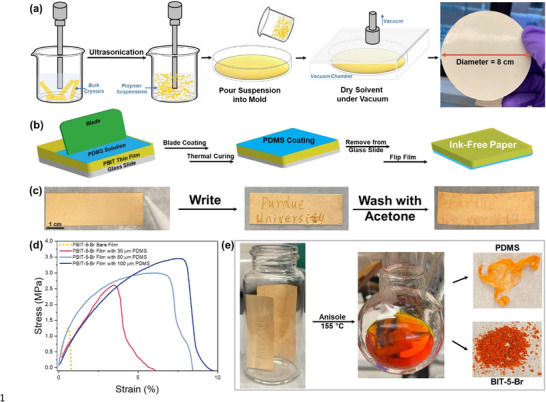
Illustration of PBIT‐PDMS composite thin film fabrications and characterizations. a) Schematic of large‐size PBIT‐5‐Br thin film fabrication via ultrasonication followed by vacuum drying, resulting in a robust, free‐standing film with an 8 cm diameter. b) Schematic of PDMS coating onto the PBIT‐5‐Br film to create ink‐free paper. c) Photographs of the PBIT‐PDMS composite film, demonstrating its writing applications. Marks made on the film can be washed off with acetone. d) Tensile stress‐strain curves of the PBIT‐5‐Br bare film and the PBIT‐5‐Br film composited with various thicknesses of PDMS. e) Recycling process of used ink‐free paper: Heating in anisole at 155 °C for 20 min allows BIT‐5‐Br powder to be easily separated from PDMS films, achieving over 95% recycling yield.

Unlike other amorphous or semicrystalline polymers that can be processed into various shapes via extrusion, injection molding, or 3D printing, PBIT polymer crystals lack effective processing methods primarily due to their poor solubility in common organic or inorganic solvents.^[^ ^40^, ^42^ ^]^ For PBIT crystals, any melt process is challenging because the melting point of PBIT is higher than the degradation temperature of the monomer.^[^ ^39^, ^40^ ^]^ The unique depolymerizable feature with color change under mechanical forces inspired us to utilize PBIT films for ink‐free paper writing applications. We first developed a method for fabricating large‐size PBIT thin films via ultrasonication of polymer crystals in solution, followed by molding the film under vacuum (**Figure** **4**a). Ultrasonication effectively disrupts the interchain interactions among PBIT polymer chains, converting bulk crystals into polymer suspensions (Figure S22, Supporting Information). The suspension was then poured into a glass petri dish, serving as the mold for film fabrication. The petri dish with PBIT suspensions was placed in a vacuum chamber, where vacuum was applied to remove solvents and smooth out the resulting thin film (Figure S23, Supporting Information). Through this process, a free‐standing and smooth PBIT thin film with a diameter of 8 cm was obtained (Figure 4a).

The thin films from crystalline PBIT polymers are inherently brittle and prone to cracking. To enhance the flexibility and durability of PBIT films, we fabricated composite PBIT films with PDMS^[^ ^60^, ^61^, ^62^, ^63^, ^64^ ^]^ using a blade coating technique (Figure 4b). PBIT‐5‐Br polymer was selected for composite film fabrication due to its highest modulus among all reported PBIT polymers.^[^ ^42^ ^]^ The blade coating procedure employed an adjustable film applicator to precisely apply a micron‐scale PDMS thin layer onto the surface of pristine PBIT thin films (Figure S24, Supporting Information). The primary objective of this coating process was to augment the mechanical robustness of the film and mitigate the likelihood of crack initiation and propagation during subsequent writing activities. Following the blade coating of PDMS thin layers on top of the PBIT film and curing at 70 °C for 12 h, a composite PBIT‐PDMS thin film with a robust PDMS backing layer was fabricated (details about the composite film fabrication can be found in Supporting Information and Video S4, Supporting Information). The film was then flipped, exposing the PDMS‐free side for writing activities. Using the tip of a mechanical pencil without graphite lead, smooth and clear writing can be achieved on the composite thin films (Figure 4c, the video about writing on PBIT‐PDMS composite thin films is attached as Video S5, Supporting Information). Unlike other ink‐free paper demonstrations that are either easily reversible and lose contrast or require complex visualization setups,^[^ ^63^, ^64^, ^65^ ^]^ the pressure applied via the pencil tip onto the composite thin film is sufficient to trigger the elongated C─C bond cleavage of PBIT, leaving permanent and irreversible marks on the film. Additionally, the depolymerized BIT monomer marks on the ink‐free composite films can be washed away with organic solvents such as acetone, allowing the films to be reused (Figure 4c).

Coating PDMS onto PBIT thin films significantly enhances their durability. PDMS is known for its viscoelasticity, exhibiting both elastic behavior and slow deformation under stress. This elasticity provides initial resistance to force. When coated on the brittle PBIT film, PDMS acts as a stress‐dispersive layer. The ultimate tensile strength significantly improved from ≈1 MPa to a maximum of 3.5 MPa with varying coating thicknesses (Figure 4d). Scanning electron microscopy (SEM) of pristine PBIT film and PBIT‐PDMS composite film clearly showed that PDMS filled the voids between PBIT crystals, resulting in an interconnected composite polymer matrix (Figure S25, Supporting Information). Upon application of tensile load on the coated films, PDMS distributed the stress more evenly across the film surface, helping to prevent localized stress concentrations that might cause the film to crack. The elastomeric properties of PDMS also helped bridge small cracks that initiated in the uncoated brittle film under tensile loading. By filling the cracks and absorbing some of the energy, PDMS potentially slowed down crack propagation and improved the film's resistance to complete fracture.

To evaluate the reusability of PBIT–PDMS ink‐free paper, we conducted multiple cycles of writing and regeneration using composite films with varying PBIT layer thicknesses. We found that thinner films (e.g., 70 µm) offered better writability due to enhanced PDMS binding, but retained visible residual traces after regeneration (Figure S26, Supporting Information). In contrast, thicker films (e.g., 140 µm) exhibited improved reusability, with residual marks effectively minimized through acetone washing and hand pressing, though at the cost of reduced writability due to increased crystallinity (Figure S27, Supporting Information). These findings highlight a tunable balance between writability and reusability, suggesting that film thickness can be optimized based on specific application needs. Additionally, coating PBIT films with PDMS does not affect the chemical composition of PBIT polymers, and the solvent‐assisted recyclability of PBIT films remains intact in the composite films. As presented in Figure 4e, used PBIT‐PDMS composite films were placed in anisole and heated at 155 °C for 20 min. Near‐quantitative depolymerization of PBIT was achieved while the PDMS layer remained intact. The resulting BIT‐5‐Br powder was easily separated from PDMS films and collected with over 95% recycling yield. The ^1^H NMR spectrum of the recycled BIT‐5‐Br monomer was identical to that of the pristine monomers (Figure S28, Supporting Information).

In summary, we investigated a novel strategy to cleave elongated C─C single bonds in polybiidenedionediyl (PBIT) derivative topochemical polymers using grinding, ball milling, and compression techniques. Rapid and efficient C─C bond cleavage and depolymerization of PBIT polymers were observed, accompanied by significant color changes. DFT calculations indicated that a force range of 4.3–5.0 nN is required to selectively cleave the elongated C─C single bonds in PBIT crystals, similar to the force needed for typical homolytic mechanophores. For polymers with lower depolymerizability, bond cleavage was found to be highly sensitive to the direction of applied mechanical stress. Studies on C─C bond cleavage and depolymerization kinetics indicate that shorter C─C bond lengths, combined with strong interchain interactions among PBIT polymer chains, can significantly increase the mechanical force required to initiate bond scission. Additionally, large‐size PBIT thin films were fabricated and composited with PDMS as a supporting back layer. These composite films were utilized as an ink‐free paper for writing and stress visualization applications. The strategy of cleaving and depolymerizing C─C bonds in PBIT polymer single crystals under mechanical stress showcases the potential of PBIT polymers as innovative mechanoresponsive materials. Furthermore, the ink‐free paper demonstration introduces a novel method to utilize topochemical polymer single crystals that are otherwise challenging to process.

## Conflict of Interest

The authors declare no conflict of interest.

## Author Contributions

Z.W., H.K., and N.H. contributed equally to this work. The project was designed by L.D. and Z.W. Z.W. carried out material synthesis, mechanical depolymerization tests, polymer characterizations, and conducted ultrasonication processing. H.K. and B.M.S. conducted CoGEF simulations. Z.W. and N.H. carried out PBIT‐PDMS composite film fabrications, ink‐free writing tests, and mechanical tests of the films. Q.H. performed SEM imaging analysis of composite films. Q.H. and X.L. helped with material synthesis and depolymerization study. L.D., Z.W., H.K., and N.H. wrote the paper with contributions from all authors. Q.H., K.M., K.W., S.Z., X.L., Y.H.L., S.Q.C., C.S.D., and B.M.S. discussed the data interpretation and commented on the paper.

## Supporting information



Supporting Information

Supplemental Video 1

Supplemental Video 2

Supplemental Video 3

Supplemental Video 4

Supplemental Video 5

## Data Availability

The data that support the findings of this study are available from the corresponding author upon reasonable request.

## References

[1] Y. Chen , G. Mellot , D. van Luijk , C. Creton , R. P. Sijbesma , Chem. Soc. Rev. 2021, 50, 4100. 33543174 10.1039/d0cs00940g

[2] M. A. Ghanem , A. Basu , R. Behrou , N. Boechler , A. J. Boydston , S. L. Craig , Y. Lin , B. E. Lynde , A. Nelson , H. Shen , D. W. Storti , Nat. Rev. Mater. 2021, 6, 84.

[3] C. M. M. Weder , J. Mater. Chem. 2011, 21, 8235.

[4] K. M. Herbert , S. Schrettl , S. J. Rowan , C. Weder , Macromolecules 2017, 50, 8845.

[5] M. M. Caruso , D. A. Davis , Q. Shen , S. A. Odom , N. R. Sottos , S. R. White , Moore, Chem. Rev. 2009, 109, 5755. 19827748 10.1021/cr9001353

[6] A. L. Black , J. M. Lenhardt , S. L. Craig , J. Mater. Chem. 2011, 21, 1655.

[7] H. Zhang , F. Gao , X. Cao , Y. Li , Y. Xu , W. Weng , R. Boulatov , Angew. Chem., Int. Ed. 2016, 55, 3040. 10.1002/anie.201510171 26805709

[8] M. J. Robb , T. A. Kim , A. J. Halmes , S. R. White , N. R. Sottos , J. S. Moore , J. Am. Chem. Soc. 2016, 138, 12328. 27616572 10.1021/jacs.6b07610

[9] M. E. McFadden , M. J. Robb , J. Am. Chem. Soc. 2019, 141, 11388. 31282668 10.1021/jacs.9b05280

[10] D. A. Davis , A. Hamilton , J. Yang , L. D. Cremar , D. Van Gough , S. L. Potisek , M. T. Ong , P. V. Braun , T. J. Martínez , S. R. White , J. S. Moore , N. R. Sottos , Nature 2009, 459, 68. 19424152 10.1038/nature07970

[11] G. R. Gossweiler , G. B. Hewage , G. Soriano , Q. Wang , G. W. Welshofer , X. Zhao , S. L. Craig , ACS Macro Lett. 2014, 3, 216. 35590509 10.1021/mz500031q

[12] Y. Sagara , M. Karman , E. Verde‐Sesto , K. Matsuo , Y. Kim , N. Tamaoki , C. Weder , J. Am. Chem. Soc. 2018, 140, 1584. 29355316 10.1021/jacs.7b12405 PMC5806082

[13] F. Verstraeten , R. Göstl , R. P. Sijbesma , Chem. Commun. 2016, 52, 8608. 10.1039/c6cc04312g 27326922

[14] Z. Chen , J. A. M. Mercer , X. Zhu , J. A. H. Romaniuk , R. Pfattner , L. Cegelski , T. J. Martinez , N. Z. Burns , Y. Xia , Science 2017, 357, 475. 28774923 10.1126/science.aan2797

[15] J. Yang , M. Horst , J. A. H. Romaniuk , Z. Jin , L. Cegelski , Y. Xia , J. Am. Chem. Soc. 2019, 141, 6479. 30969109 10.1021/jacs.9b01736

[16] N. Frickel , R. Messing , A. M. Schmidt , J. Mater. Chem. 2011, 21, 8466.

[17] D.‐H. Kim , P. Karavayev , E. A. Rozhkova , J. Pearson , V. Yefremenko , S. D. Bader , V. Novosad , J. Mater. Chem. 2011, 21, 8422.

[18] M. B. Larsen , A. J. Boydston , J. Am. Chem. Soc. 2013, 135, 8189. 23687904 10.1021/ja403757p

[19] H. Zhang , Y. Chen , Y. Lin , X. Fang , Y. Xu , Y. Ruan , W. Weng , Macromolecules 2014, 47, 6783.

[20] Z. Xia , V. D. Alphonse , D. B. Trigg , T. P. Harrigan , J. M. Paulson , Q. T. Luong , E. P. Lloyd , M. H. Barbee , S. L. Craig , Molecules 2019, 24, 542. 30717294 10.3390/molecules24030542 PMC6384768

[21] T.‐G. Hsu , J. Zhou , H.‐W. Su , B. R. Schrage , C. J. Ziegler , J. Wang , J. Am. Chem. Soc. 2020, 142, 2100. 31940198 10.1021/jacs.9b12482

[22] Y. Lin , T. B. Kouznetsova , S. L. Craig , J. Am. Chem. Soc. 2020, 142, 2105. 31939656 10.1021/jacs.9b13359

[23] J. Zhou , T.‐G. Hsu , J. Wang , Angew. Chem., Int. Ed. 2023, 62, 202300768.

[24] J. Li , C. Nagamani , J. S. Moore , Acc. Chem. Res. 2015, 48, 2181. 26176627 10.1021/acs.accounts.5b00184

[25] G. A. Filonenko , J. R. Khusnutdinova , Adv. Mater. 2017, 29, 1700563. 10.1002/adma.201700563 28318067

[26] G. B. Schuster , N. J. Turro , H. C. Steinmetzer , A. P. Schaap , G. Faler , W. Adam , J. C. Liu , J. Am. Chem. Soc. 1975, 97, 7110.

[27] J. Park , Y. Lee , M. H. Barbee , S. Cho , S. Cho , R. Shanker , J. Kim , J. Myoung , M. P. Kim , C. Baig , S. L. Craig , H. Ko , Adv. Mater. 2019, 31, 1808148. 10.1002/adma.201808148 31070272

[28] M. Raisch , D. Genovese , N. Zaccheroni , S. B. Schmidt , M. L. Focarete , M. Sommer , C. Gualandi , Adv. Mater. 2018, 30, 1802813. 10.1002/adma.201802813 30133005

[29] Y.‐K. Song , K.‐H. Lee , W.‐S. Hong , S.‐Y. Cho , H.‐C. Yu , C.‐M. Chung , J. Mater. Chem. 2012, 22, 1380.

[30] J. Li , T. Shiraki , B. Hu , R. A. E. Wright , B. Zhao , J. S. Moore , J. Am. Chem. Soc. 2014, 136, 15925. 25360903 10.1021/ja509949d

[31] M. Karman , E. Verde‐Sesto , C. Weder , Y. C. Simon , ACS Macro Lett. 2018, 7, 1099. 35632942 10.1021/acsmacrolett.8b00591

[32] Y. Chen , A. J. H. Spiering , S. Karthikeyan , G. W. M. Peters , E. W. Meijer , R. P. Sijbesma , Nat. Chem 2012, 4, 559. 22717441 10.1038/nchem.1358

[33] Y. Sun , W. J. Neary , Z. P. Burke , H. Qian , L. Zhu , J. S. Moore , J. Am. Chem. Soc. 2022, 144, 1125. 35019277 10.1021/jacs.1c12108

[34] Y. Sun , K. Wang , X. Huang , S. Wei , E. Contreras , P. K. Jain , L. M. Campos , H. J. Kulik , J. S. Moore , J. Am. Chem. Soc. 2024, 146, 27117. 39306733 10.1021/jacs.4c09926

[35] B. A. Beiermann , D. A. Davis , S. L. B. Kramer , J. S. Moore , N. R. Sottos , S. R. White , J. Mater. Chem. 2011, 21, 8443.

[36] H. Oka , K. Imato , T. Sato , T. Ohishi , R. Goseki , H. Otsuka , ACS Macro Lett. 2016, 5, 1124. 35658193 10.1021/acsmacrolett.6b00529

[37] N. A. A. Rossi , E. J. Duplock , J. Meegan , D. R. T. Roberts , J. J. Murphy , M. Patel , S. J. Holder , J. Mater. Chem. 2009, 19, 7674.

[38] J. W. Woodcock , R. Beams , C. S. Davis , N. Chen , S. J. Stranick , D. U. Shah , F. Vollrath , J. W. Gilman , Adv. Mater. Interfaces 2017, 4, 1601018. 10.1002/admi.201601018 PMC8240949 34194923

[39] L. Dou , Y. Zheng , X. Shen , G. Wu , K. Fields , W.‐C. Hsu , H. Zhou , Y. Yang , F. Wudl , Science 2014, 343, 272. 24436414 10.1126/science.1245875

[40] X. Luo , Z. Wei , B. Seo , Q. Hu , X. Wang , J. A. Romo , M. Jain , M. Cakmak , B. W. Boudouris , K. Zhao , J. Mei , B. M. Savoie , L. Dou , J. Am. Chem. Soc. 2022, 144, 16588. 35994519 10.1021/jacs.2c06417

[41] Q. Hu , X. Luo , L. A. Ogunfowora , A. Athaley , J. S. DesVeaux , B. C. Klein , S. Xu , P. Wu , Z. Wei , C. Lin , et al., Nat. Chem. Eng. 2025, 2, 130.

[42] Z. Wei , X. Wang , B. Seo , X. Luo , Q. Hu , J. Jones , M. Zeller , K. Wang , B. M. Savoie , K. Zhao , L. Dou , Angew. Chem., Int. Ed. 2022, 61, 202213840. 10.1002/anie.202213840 PMC10092176 36219546

[43] L. Long , S. Medina Rivero , F. Sun , D. Wang , D. Chekulaev , C. Tonnelé , D. Casanova , J. Casado , Y. Zheng , Angew. Chem., Int. Ed. 2023, 62, 202308780. 10.1002/anie.202308780 37533303

[44] M. K. Beyer , J. Chem. Phys. 2000, 112, 7307.

[45] S. Grimme , S. Ehrlich , L. Goerigk , J. Comput. Chem. 2011, 32, 1456. 21370243 10.1002/jcc.21759

[46] T. Stauch , A. Dreuw , Chem. Rev. 2016, 116, 14137. 27767298 10.1021/acs.chemrev.6b00458

[47] B. Fanconi , J. F. Rabolt , J. Polym. Sci. Polym. Phys. Ed. 1985, 23, 1201.

[48] K. Tashiro , G. Wu , M. Kobayashi , Polymer 1988, 29, 1768.

[49] K. Tashiro , Prog. Polym. Sci. 1993, 18, 377.

[50] K. Wasanasuk , K. Tashiro , Macromolecules 2012, 45, 7019.

[51] T. Kurita , Y. Fukuda , M. Takahashi , Y. Sasanuma , ACS Omega 2018, 3, 4824. 31458699 10.1021/acsomega.8b00506 PMC6641976

[52] S. Xu , J. Zhou , P. Pan , Prog. Polym. Sci. 2023, 140, 101676.

[53] C. Nagamani , H. Liu , J. S. Moore , J. Am. Chem. Soc. 2016, 138, 2540. 26895404 10.1021/jacs.6b00097

[54] M. B. Gordon , S. Wang , G. A. Knappe , N. J. Wagner , T. H. Epps , C. J. Kloxin , Polym. Chem. 2017, 8, 6485.

[55] Z. Chen , X. Zhu , J. Yang , J. A. M. Mercer , N. Z. Burns , T. J. Martinez , Y. Xia , Nat. Chem 2020, 12, 302. 31907403 10.1038/s41557-019-0396-5

[56] H. Sakai , T. Sumi , D. Aoki , R. Goseki , H. Otsuka , ACS Macro Lett. 2018, 7, 1359. 35651243 10.1021/acsmacrolett.8b00755

[57] B. Lee , Z. Niu , J. Wang , C. Slebodnick , S. L. Craig , J. Am. Chem. Soc. 2015, 137, 10826. 26247609 10.1021/jacs.5b06937

[58] J. Wang , T. B. Kouznetsova , S. L. Craig , J. Am. Chem. Soc. 2016, 138, 10410. 27500711 10.1021/jacs.6b06452

[59] H. M. Klukovich , Z. S. Kean , S. T. Iacono , S. L. Craig , J. Am. Chem. Soc. 2011, 133, 17882. 21967190 10.1021/ja2074517

[60] P. Siribunbandal , T. Osotchan , Y.‐H. Kim , R. Jaisutti , ACS Appl. Polym. Mater. 2023, 5, 7786.

[61] D.‐H. Park , J. Hong , I. S. Park , C. W. Lee , J.‐M. Kim , Adv. Funct. Mater. 2014, 24, 5186.

[62] P. Zhang , X. Shi , A. P. H. J. Schenning , G. Zhou , L. T. de Haan , Adv. Mater. Interfaces 2020, 7, 1901878.

[63] V. Müller , T. Hungerland , M. Baljozovic , T. Jung , N. D. Spencer , H. Eghlidi , P. Payamyar , A. D. Schlüter , Adv. Mater. 2017, 29, 1701220. 10.1002/adma.201701220 28485053

[64] Y. Xie , Y. Meng , W. Wang , E. Zhang , J. Leng , Q. Pei , Adv. Funct. Mater. 2018, 28, 1802430.

[65] W. Wang , N. Xie , L. He , Y. Yin , Nat. Commun 2014, 5, 5459. 25463000 10.1038/ncomms6459

