# Supplementary material for: Force‐Induced Selective Carbon‐Carbon Bond Cleavage in Mechanoresponsive Topochemical Polymers
Source: Adv Mater. 2025 Sep 18;38(2):e10482. doi: 10.1002/adma.202510482 (PMC12783939; doi:10.1002/adma.202510482)
Supplement: Supplementary file 1 — Supporting Information [file ADMA-38-e10482-s002.docx]

Supporting Information

**Force-induced Selective Carbon-Carbon Bond Cleavage in Mechanoresponsive Topochemical Polymers**

*Zitang Wei^1,2^,^†,^* Hanul Kim^1,3^,^†^ Nazmul Haque^4^,^†^ Qixuan Hu^1^, Ke Ma^1,5^, Kang Wang^1,6^, Shuchen Zhang^1,7^, Xuyi Luo^1,8^, Yoon Ho Lee^1,9^, Siyoung Q. Choi^3^, Chelsea S. Davis^4,10^, Brett M. Savoie^1,11^*, and Letian Dou^1,12,13^**

^1^Davidson School of Chemical Engineering, Purdue University, West Lafayette, IN 47907, USA

^2^Department of Chemical Engineering, Massachusetts Institute of Technology, Cambridge, MA 02139, USA

^3^Department of Chemical and Biomolecular Engineering, Korea Advanced Institute of Science and Technology (KAIST), Daejeon, 34141, Republic of Korea

^4^School of Materials Engineering, Purdue University, West Lafayette, IN 47907, USA

^5^Global Institute of Future Technology, Shanghai Jiao Tong University, Shanghai, 200240, China

^6^Key Laboratory of Photochemistry, Institute of Chemistry, Chinese Academy of Sciences, Beijing, 100190, China.

^7^State Key Laboratory of Precision and Intelligent Chemistry, Department of Materials Science and Engineering, School of Chemistry and Materials Science, University of Science and Technology of China, Hefei, 230026, China

^8^Department of Chemistry, Stanford University, Stanford, CA, 94305, USA

^9^Department of Materials Science & Engineering, Sungshin Women’s University, Seoul, 01133, Republic of Korea

^10^Department of Mechanical Engineering, University of Delaware, Newark, DE 19716, USA

^11^Department of Chemical and Biomolecular Engineering, University of Notre Dame, Notre Dame, IN 46556, USA

^12^Department of Chemistry, Purdue University, West Lafayette, IN 47907, USA

^13^Department of Chemistry, Emory University, Atlanta, GA 30322, USA

^†^Z.W., H.K., and N.H. contributed equally to this paper

*Corresponding authors: letian.dou@emory.edu; [bsavoie2@nd.edu](mailto:bsavoie2@nd.edu); ztwei@mit.edu

**Table of Contents**

[1. Methods 2](#_Toc191860270)

[1.1. Materials and Characterizations 2](#_Toc191860271)

[1.2. BIT Monomer Crystallization and Topochemical Polymerization 2](#_Toc191860272)

[1.3. Mechanical Depolymerization Process via Grinding 2](#_Toc191860273)

[1.4. Mechanical Depolymerization Process via Ball Milling 3](#_Toc191860274)

[1.5. Density Functional Theory (DFT) Calculations 3](#_Toc191860275)

[1.6. PBIT Film Fabrication for Hard Press Testing 3](#_Toc191860276)

[1.7. Hard Press Testing Procedure on PBIT Thin Films 3](#_Toc191860277)

[1.8. Calculations of Weight Loss Percentage of PBIT Films after Hard Press 4](#_Toc191860278)

[1.9. Large Size PBIT-5-Br Thin Film Fabrications 4](#_Toc191860279)

[1.10. PBIT-PDMS Composite Film Fabrication Process for Ink-free Paper 4](#_Toc191860280)

[2. Supplementary Figures 5](#_Toc191860281)

1. **Methods**
   1. **Materials and Characterizations**

All reagents were purchased from suppliers including Fisher Scientific, Sigma-Aldrich, VWR, and Enamine and used without further purifications. ^1^H NMR spectra were collected using a Bruker ARX 400 spectrometer where the samples were dissolved in deuterated chloroform at 298 K. UV-Vis absorption spectra were recorded on an Agilent UV-Vis-NIR Cary-5000 spectrometer in transmission mode. Single crystals were analyzed using a Bruker Quest diffractometer with kappa geometry, an I-μ-S microsource X-ray tube (Cu Kα radiation, λ = 1.54178 Å), laterally graded multilayer (Goebel) mirror for monochromatization, and a Photon2 CMOS area detector. The instrument is equipped with Oxford Cryosystems low temperature device. The examination and data collection were performed at 150 K. Ultra-sonication treatment of polymer crystals was conducted using QSonica Q700 Sonicator with a 1/2” standard probe. Ball milling on PBIT polymers was performed by MSE PRO 4L (4 x 1L) Vertical High Energy Planetary Ball Mill (Produce Number MA0142). Initial qualitative hard pressing tests on PBIT crystals were conducted with Dulytek® Elite DE10K Hybrid Rosin Heat Press, 5 Tons. Quantitative hard press testing on PBIT thin films were conducted using Mechanical Testing System (MTS) Criterion Model 43. Scanning electron microscopy (SEM) was conducted using Quanta 3D FEG. Tensile testing on PBIT-PDMS composite thin films was conducted on Load frame (µTS, Psylotech).

- 1. **BIT Monomer Crystallization and Topochemical Polymerization**

BIT monomer synthesis was conducted according to the literature.^1-3^ In a 500 mL beaker, 500 mg of BIT monomers were added, followed by 50 mL of methanol and 50 mL of dichloromethane (DCM). The beaker was covered with aluminum foil to avoid unwanted polymerization. The foil was perforated to facilitate solvent evaporation. The beaker was put in the fume hood overnight until all the solvent in the beaker was evaporated. Then monomer crystals were collected and put under a strong industrial white lamp (Global Industrial™ LED Flood Light, 50W, 4500 Lumens, 5000K). After 30 minutes of illumination, the red monomer crystals were polymerized and converted into light yellow polymer crystals.

- 1. **Mechanical Depolymerization Process via Grinding**

Grinding of PBIT polymers was conducted using a Coors™ porcelain pestle (Sigma Aldrich). In a mortar, 100 mg of PBIT polymers were placed, and continuous grinding with the pestle was applied for one minute. The residue was then rinsed with acetone and subjected to vacuum filtration to separate the depolymerized monomers from the polymers. The remaining polymer particles were returned to the mortar, and another one-minute grinding was applied. This grinding and washing process was repeated five times until the polymer residues were too small to be effectively ground. The filtered BIT monomer in acetone was dried, collected, and weighed to calculate the depolymerization yield. The purity of the depolymerized monomer was tested via ^1^H NMR and compared with pristine BIT monomers.

- 1. **Mechanical Depolymerization Process via Ball Milling**

Ball milling on PBIT polymers was conducted using MSE PRO 4L (4 x 1L) Vertical High Energy Planetary Ball Mill (Product Number MA0142) connected to an industrial chiller for consistent cooling at 20 ºC. MSE PRO 1L (1,000 ml) Teflon Planetary Ball Mill Grinding Jar and MSE PRO 2.5 mm Alumina Milling Media Balls were used for the depolymerization process. 500 mg of PBIT polymer was put in the 1-liter jar together with around 250 g of Alumina Milling Media Balls. The chiller maintained the temperature at 20 ºC during the entire milling process. The milling speed was set at 200 rpm for 20 minutes. The depolymerization yield after ball milling was around 20%.

- 1. **Density Functional Theory (DFT) Calculations**

Density Functional Theory (DFT) calculations were used to relax molecule geometry and calculate its energy in CoGEF simulation.^4^ All calculations were performed using the Gaussian16^5^ at the B3LYP/6-31G* level.^6^ The preliminary geometry of the unconstrained molecule was obtained through CREST^7^ and subsequently optimized at an equivalent DFT level in theory. Starting from the equilibrium, the initial distance (*d*_0_) between the terminal anchor atoms of the dimer structure is raised by 0.05 Å, and the energy at each step is minimized. These cycles of recurrent stretching and relaxation could be successively carried out utilizing the iterative source code that was formulated in reference.^8^ The iteration is finished after the C-C bond scission or the first reaction (for set B).

- 1. **PBIT Film Fabrication for Compression Testing**

200 mg of PBIT-5-Br crystals were mixed with chloroform (35 mL) in a 50 mL beaker. The ultrasonication probe was then immersed into the solvent. Ultrasonication parameters were set as follows: amplitude 15, 4 sec pulse and 1 sec rest. The mixture was cooled by a salt ice bath during the entire 1-hour ultrasonication processing. After sonication, the suspension was filtered with a vacuum filtration apparatus and a nylon membrane filter (Cytiva Nylon Membrane Filters 0.45μm Diameter 47 mm. Product number: 7404-004) yielding free-standing polymer thin films. Each thin film is about 50 mg and 100 µm thick.

- 1. **Compression Testing Procedure on PBIT Thin Films**

Mechanical Testing System (MTS) was used for compression testing. Before testing, the pristine PBIT films were put between two pieces of weighing paper and located on the MTS Load Cell with 50 kN capacity (Model LPS.504). Constant compression forces were applied on films for 60 seconds. Photographs of pristine and compressed films were taken to observe color changes on the films.

- 1. **Calculations of Weight Loss Percentage of PBIT Films after Hard Pressing**

The weights of pristine films were measured and recorded before compression tests on MTS setup. After the compression, the film was shredded and put in a 20 mL glass vial. Then 20 mL of dichloromethane (DCM) was added in the vial and the solution was stirred overnight to ensure complete dissolution of depolymerized monomers in the solution. After stirring, the solution was vacuum filtered, and the polymer residual was dried and weighed to calculate the weight loss during hard pressing. The UV-Vis absorption of the collected depolymerized BIT in DCM solution was measured.

- 1. **Large Size PBIT-5-Br Thin Film Fabrications**

300 mg of PBIT-5-Br crystals were mixed with chloroform (35 mL) in a 50 mL beaker. The ultrasonication probe was then immersed into the solvent. Ultrasonication parameters were set as follows: amplitude 15, 4 sec pulse and 1 sec rest. The mixture was cooled by a salt ice bath during the entire 1-hour ultrasonication processing. Extended ultrasonication times result in more brittle films. And the resulting suspension was poured into a glass petri dish (VWR® Petri Dishes, Glass with 10 mm diameter). Then the petri dish with suspension was put in an acrylic vacuum chamber connected to the vacuum and the solvent was dried overnight under vacuum. Lastly, the petri dish was taken out of the vacuum chamber and ethanol was added into the petri dish. The film floated on the ethanol and removed from the petri dish.

- 1. **PBIT-PDMS Composite Film Fabrication Process for Ink-free Paper**

An adjustable film applicator was used in the blade coating procedure to precisely apply a micron-scale thin layer of polydimethylsiloxane (PDMS) onto the reverse side of our ink-free paper substrate. Initially, manual height adjustment screws on the applicator were utilized to tailor the thickness of the PDMS layer based on specific requirements for different film thicknesses. To ensure a smooth coating process and prevent tearing, the paper was strategically positioned on top of a glass slide. The glass slide, with the paper, was then positioned beneath the applicator blade, and the blade height was adjusted carefully to achieve the desired film thickness. Subsequently, a PDMS layer was delicately placed onto the paper surface, ensuring bubble-free applications. The paper, along with the glass slide, was then moved horizontally under the blade, allowing the PDMS layer to be uniformly spread over the paper at the specified thickness corresponding to the adjusted blade height. Excess PDMS at the edges of the paper was removed to prevent any seepage between the paper and the glass slide. The coated film was subjected to curing at a temperature of 70 ºC for a duration of 12 hours. This systematic process enabled easy removal of the paper from the glass slide after the PDMS curing, leaving a robust PDMS film coating on one surface of the paper. The resulting substrate with an ink-free, robust back layer of PDMS film was employed for subsequent inkless writing experiments.

1. **Supplementary Figures**


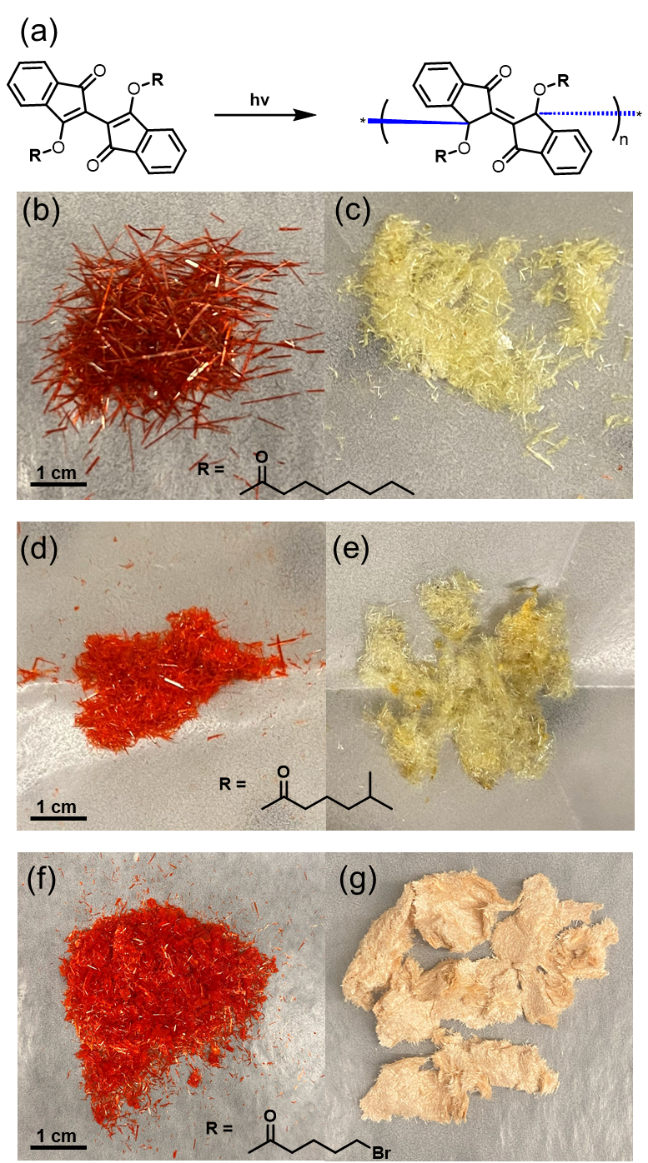


**Figure S1**. (**a**) Synthetic route of PBIT polymers. Quantitative polymerization was achieved via illumination under a strong industrial white lamp for 30 minutes. Images of BIT-8 monomer crystals (**b**) and PBIT-8 polymer crystals (**c**). Inset is the side chain of PBIT-8. Images of BIT-6-Me monomer crystals (**d**) and PBIT-6-Me polymer crystals (**e**). Inset is the side chain of PBIT-6-Me. Images of BIT-5-Br monomer crystals (**f**) and PBIT-5-Br polymer crystals (**g**). Inset is the side chain of PBIT-5-Br.


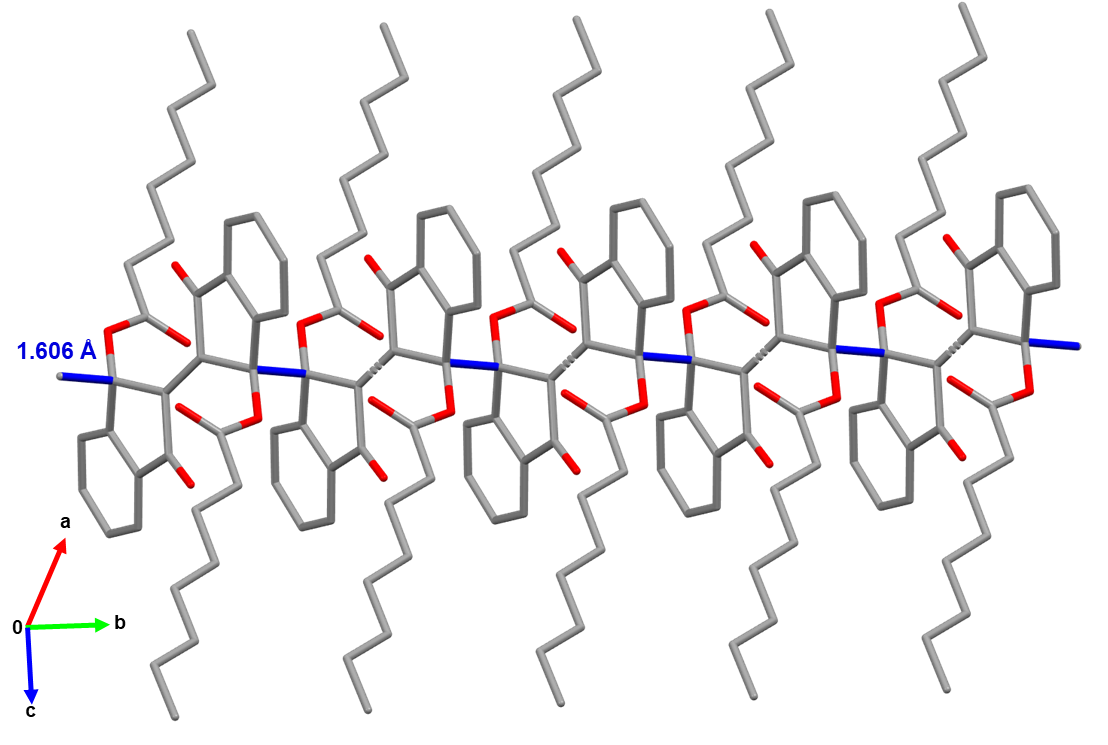


**Figure S2**. Single crystal structure of PBIT-8. Elongated carbon-carbon single bonds with bond length of 1.606 Å are highlighted in blue. Hydrogen atoms are omitted for clarity (CCDC number of PBIT-8: 2088148).


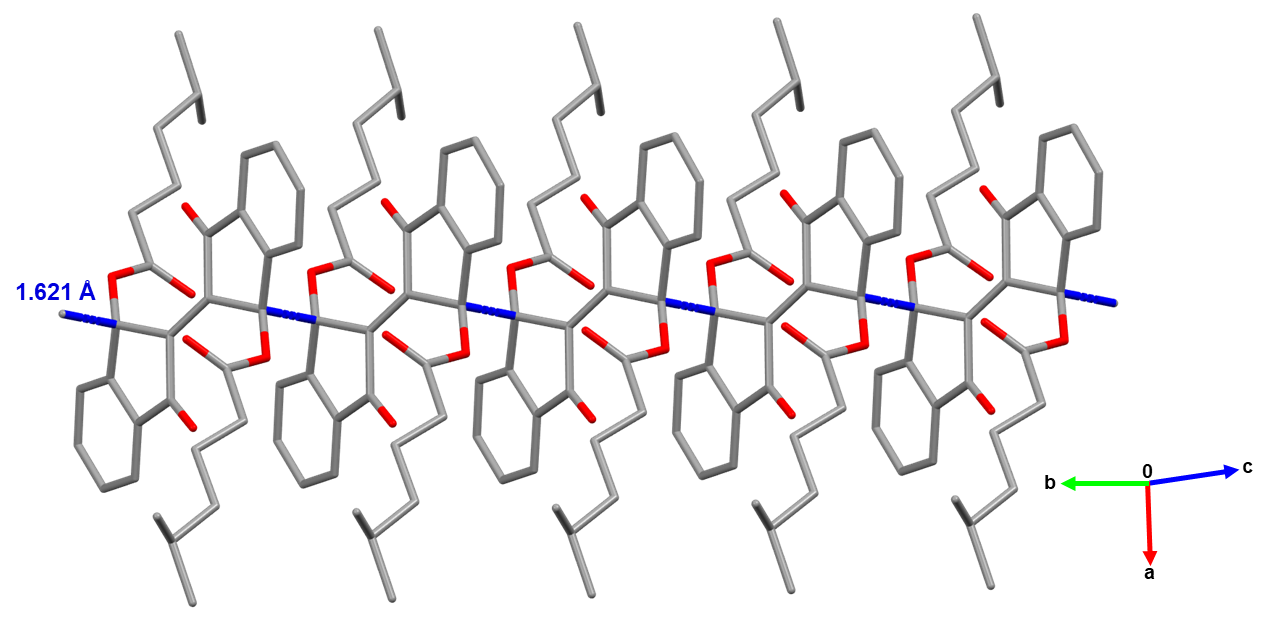


**Figure S3**. Single crystal structure of PBIT-6-Me. Elongated carbon-carbon single bonds with bond length of 1.621 Å are highlighted in blue. Hydrogen atoms are omitted for clarity. (CCDC number of PBIT-6-Me: 2131645).


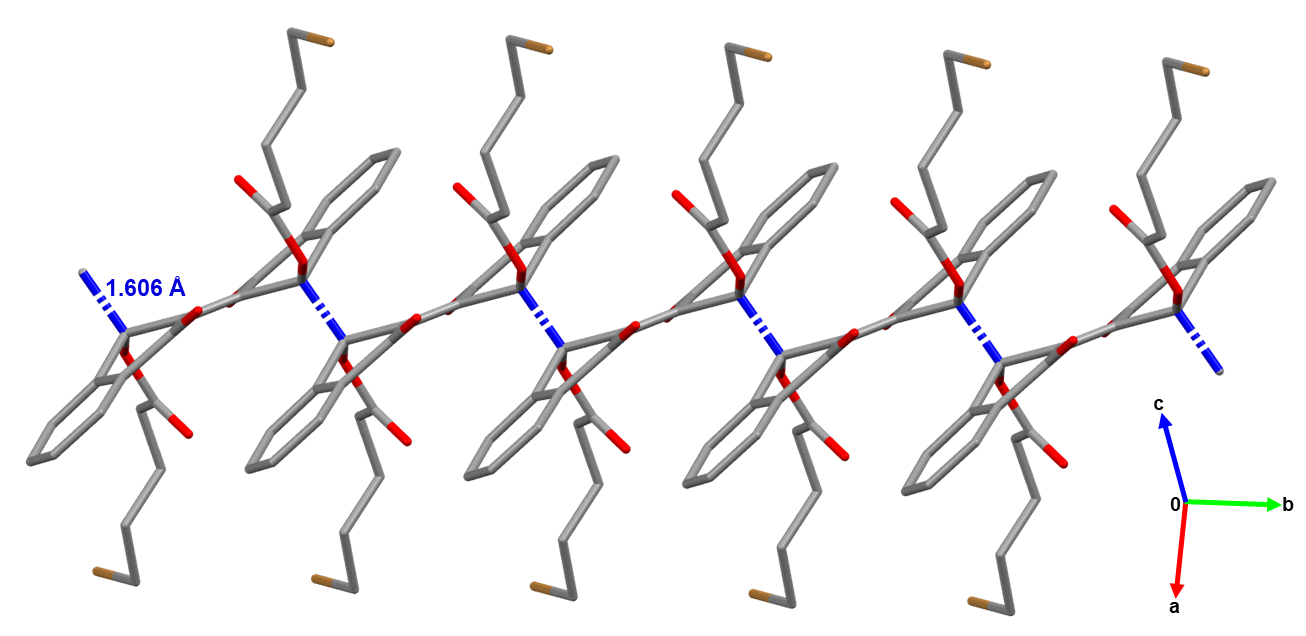


**Figure S4**. Single crystal structure of PBIT-5-Br. Elongated carbon-carbon single bonds with bond length of 1.606 Å are highlighted in blue. Hydrogen atoms are omitted for clarity. (CCDC number of PBIT-5-Br: 2177487).


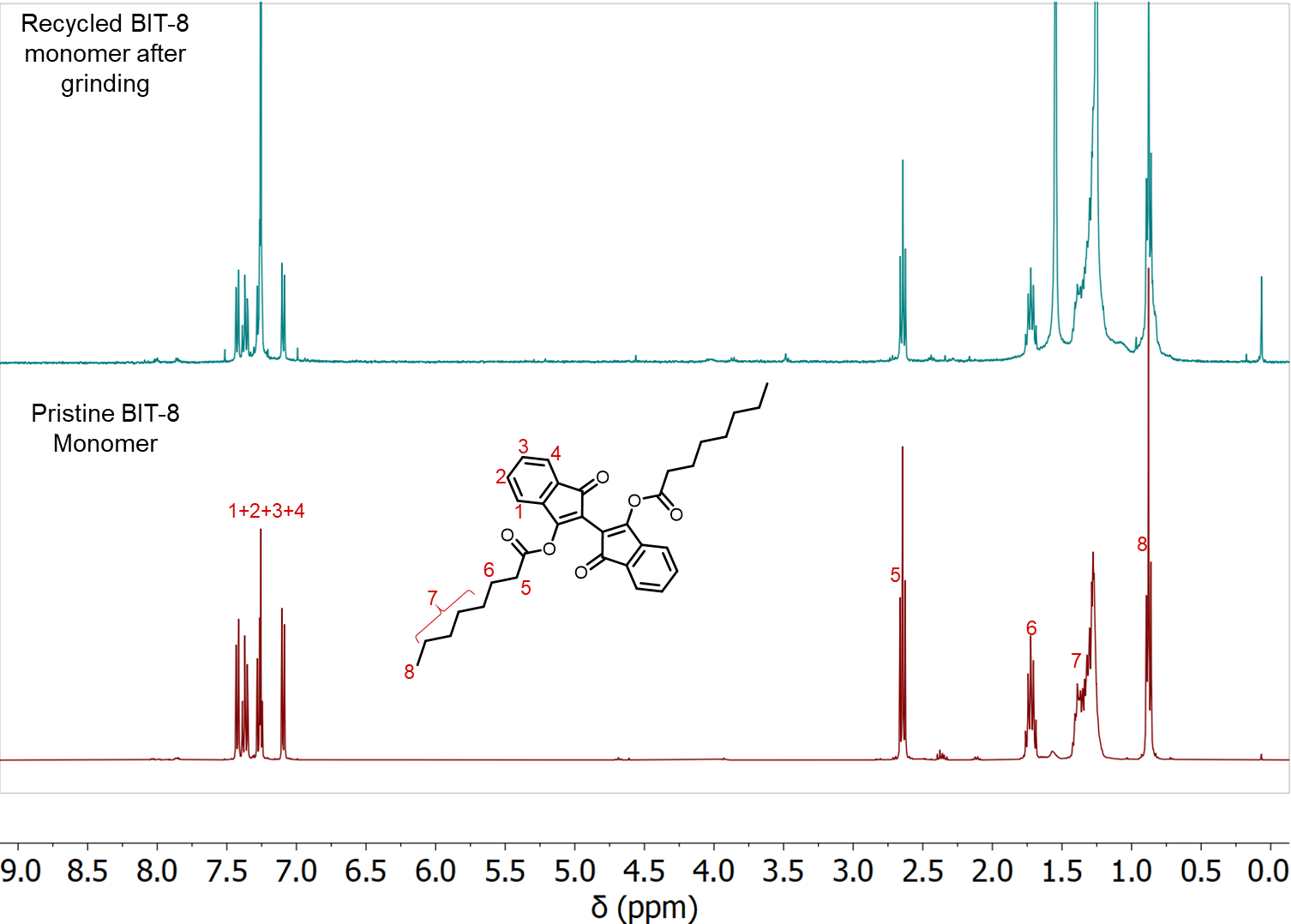


**Figure S5**. Overlays of ^1^H NMR spectra of recycled BIT-8 monomers after grinding (top) and pristine monomers (bottom) (25°C, CDCl_3_, with residual solvent peaks at 7.26, 2.17, and 1.56 ppm for CHCl_3_, acetone, and H_2_O, respectively).


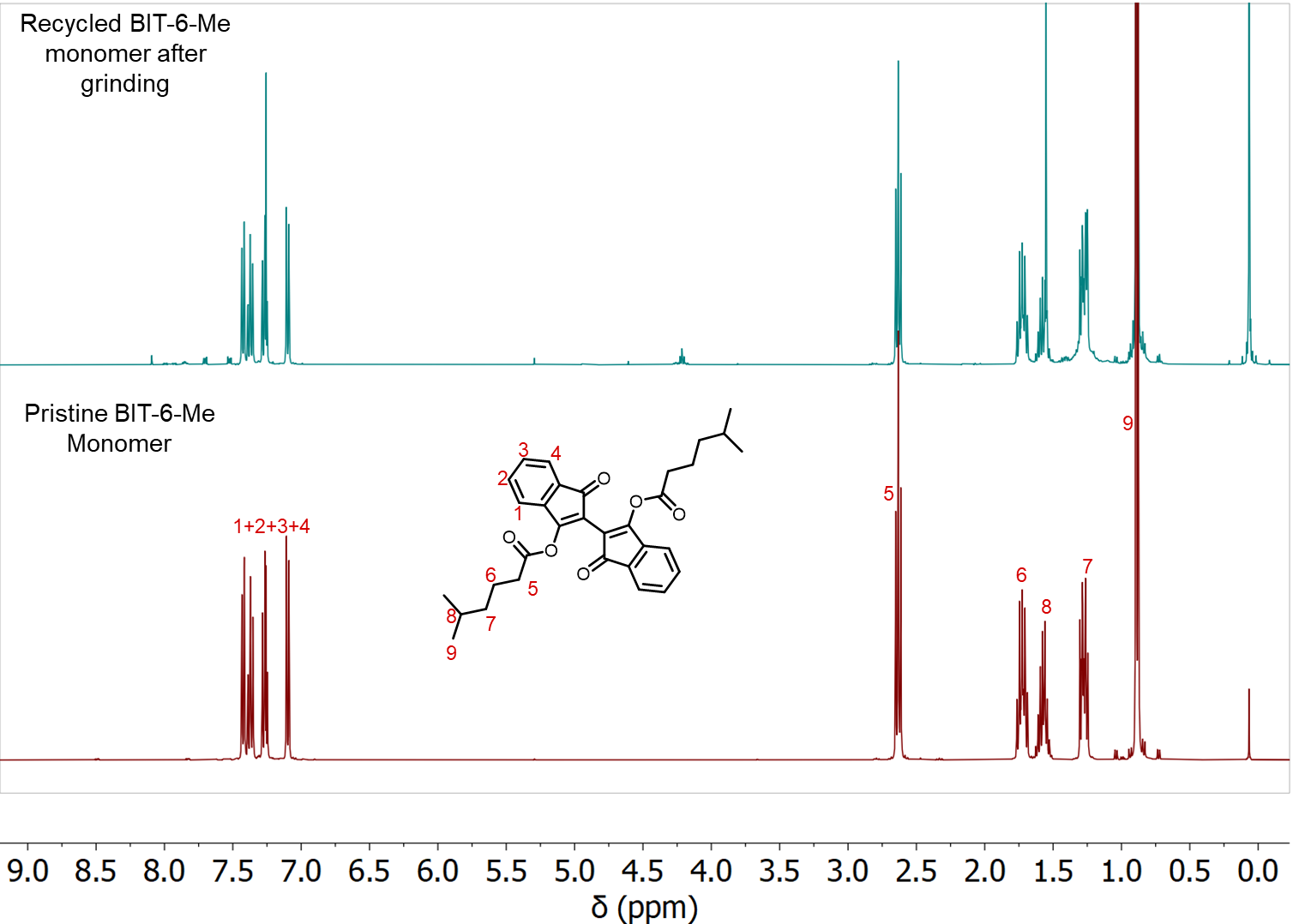


**Figure S6**. Overlays of ^1^H NMR spectra of recycled BIT-6-Me monomers after grinding (top) and pristine monomers (bottom) (25°C, CDCl_3_, with residual solvent peaks at 7.26, 2.17, and 1.56 ppm for CHCl_3_, acetone, and H_2_O, respectively).


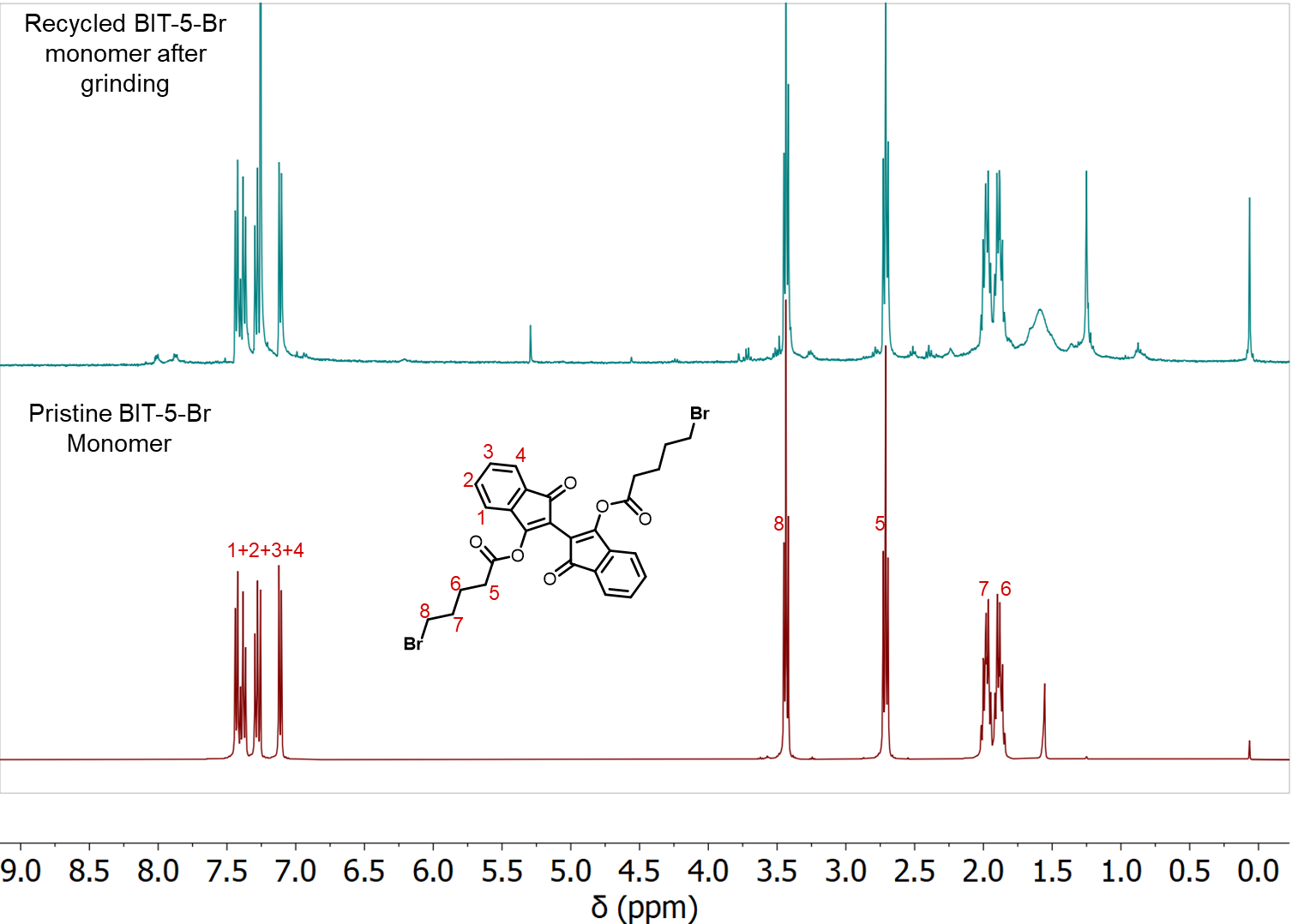


**Figure S7**. Overlays of ^1^H NMR spectra of recycled BIT-5-Br monomers after grinding (top) and pristine monomers (bottom) (25°C, CDCl_3_, with residual solvent peaks at 7.26, 2.17, and 1.56 ppm for CHCl_3_, acetone, and H_2_O, respectively).


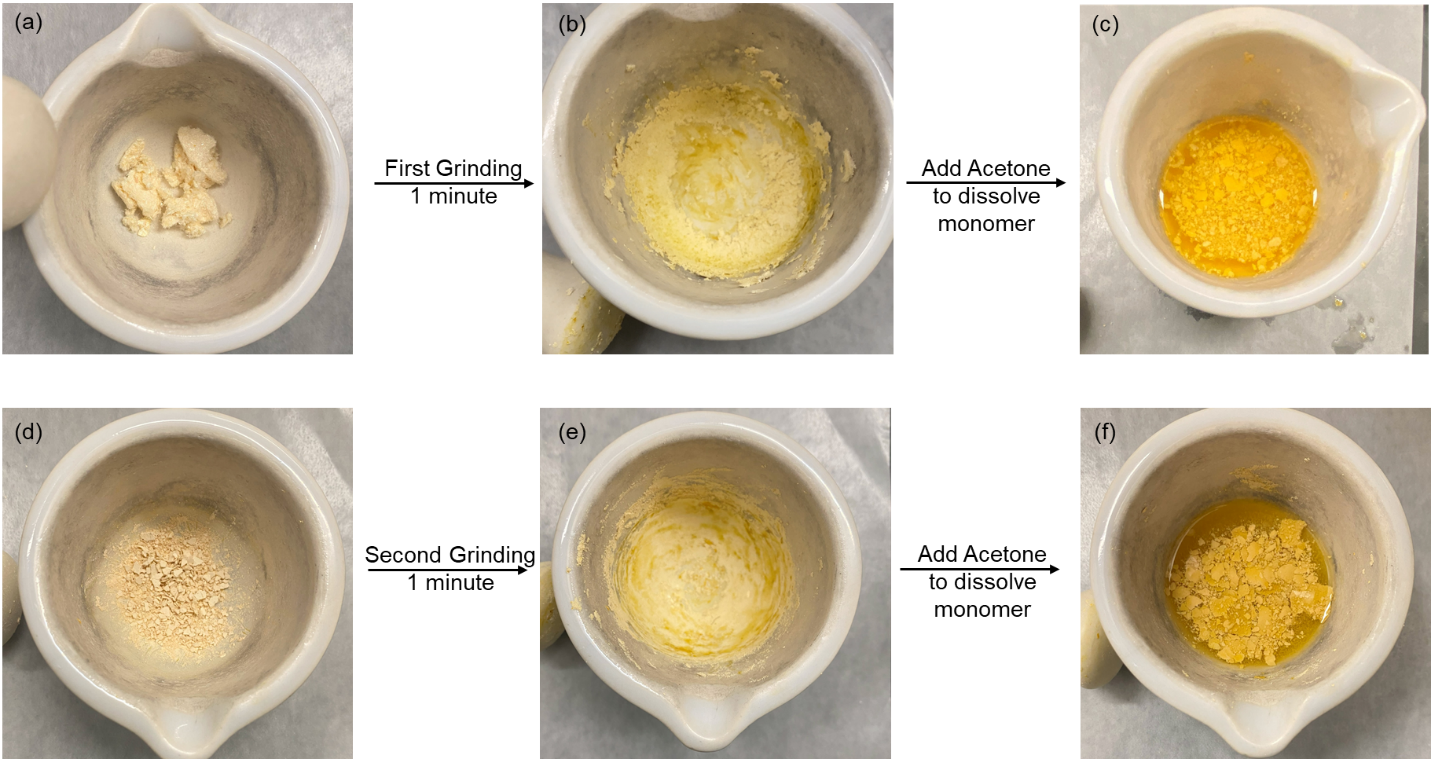


**Figure S8**. Illustration of multiple grinding on PBIT polymer crystals to enhance mechanical depolymerization yield. After 100 mg PBIT-8 polymers were put in the mortar (**a**), first grinding was applied via hand grinding with a pestle for one minute and color change was observed from light yellow to orange (**b**). Then 10 mL of acetone was added into the mortar to dissolve depolymerized BIT-8 monomers (**c**), followed by vacuum filtration to remove the solution. The remaining PBIT-8 polymers were put again into the mortar (**d**), and the second grinding was applied for one minute (**e**). Acetone was added again after the hand grinding and the mixture was filtered to repeat the grinding process (**f**). Five consecutive grindings were done on those PBIT-8 polymers to achieve a depolymerization yield of 60%.


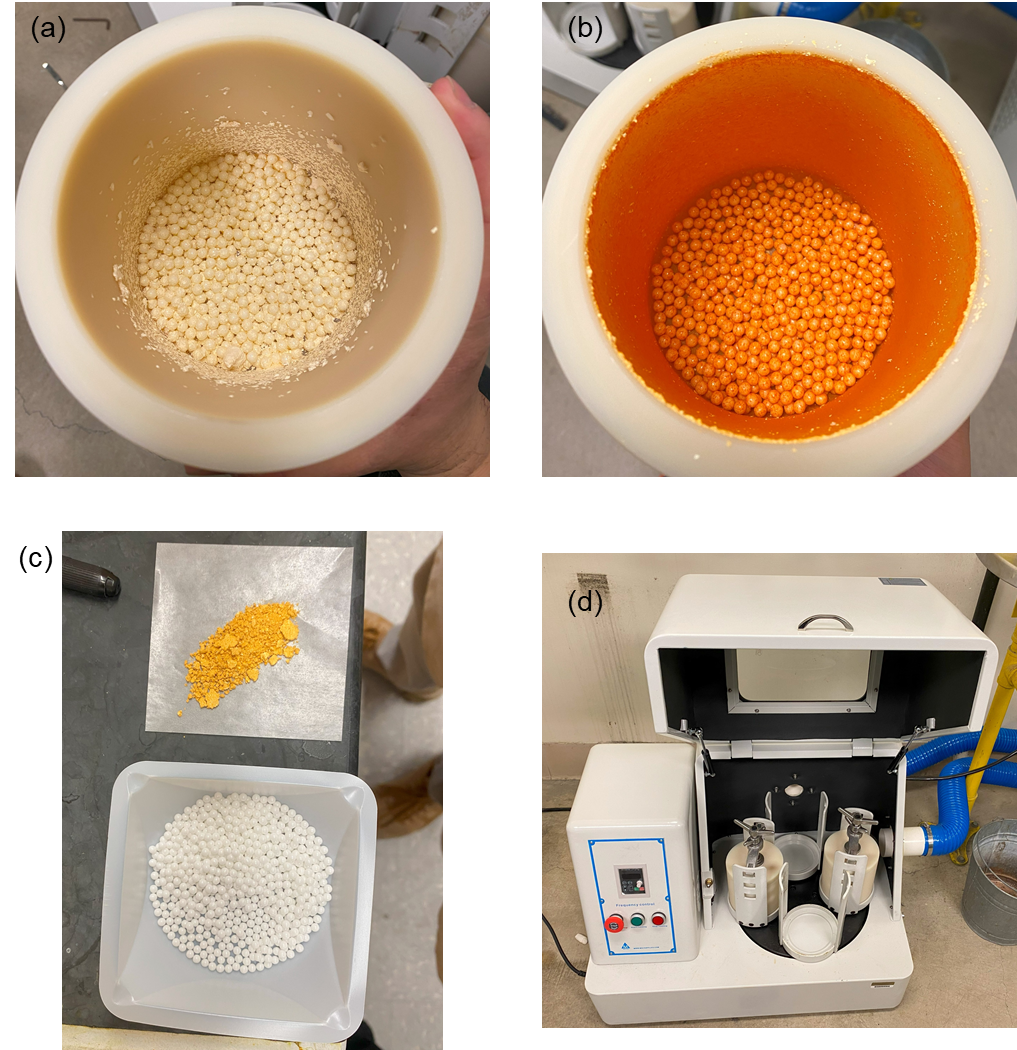


**Figure S9**. Mechanical depolymerization of PBIT polymers using ball milling setup. (**a**) PBIT polymer with 2.5 mm Alumina Milling Media Balls in the Ball Mill Grinding Jar before milling. (**b**) PBIT polymer with 2.5 mm Alumina Milling Media Balls in the Ball Mill Grinding Jar after milling. (**c**) Separated PBIT polymer residue after ball milling (top) from Alumina Milling Media Balls (bottom). (**d**) Ball mill setup. Blue tubes were connected to an industrial chiller cooled at 20 ºC during the entire process. It is noteworthy that compared to hand grinding and hard pressing, which typically process only 10–100 mg of PBIT polymer, ball milling enables gram-scale treatment. In the experiment shown here, 2 g of PBIT-8 were subjected to ball milling, resulting in a visibly more intense orange coloration of the jar surface. Although similar depolymerization yield can be reached for both hand grinding and ball milling methods, the stronger color observed in ball milling is attributed to greater monomer accumulation and surface coverage.


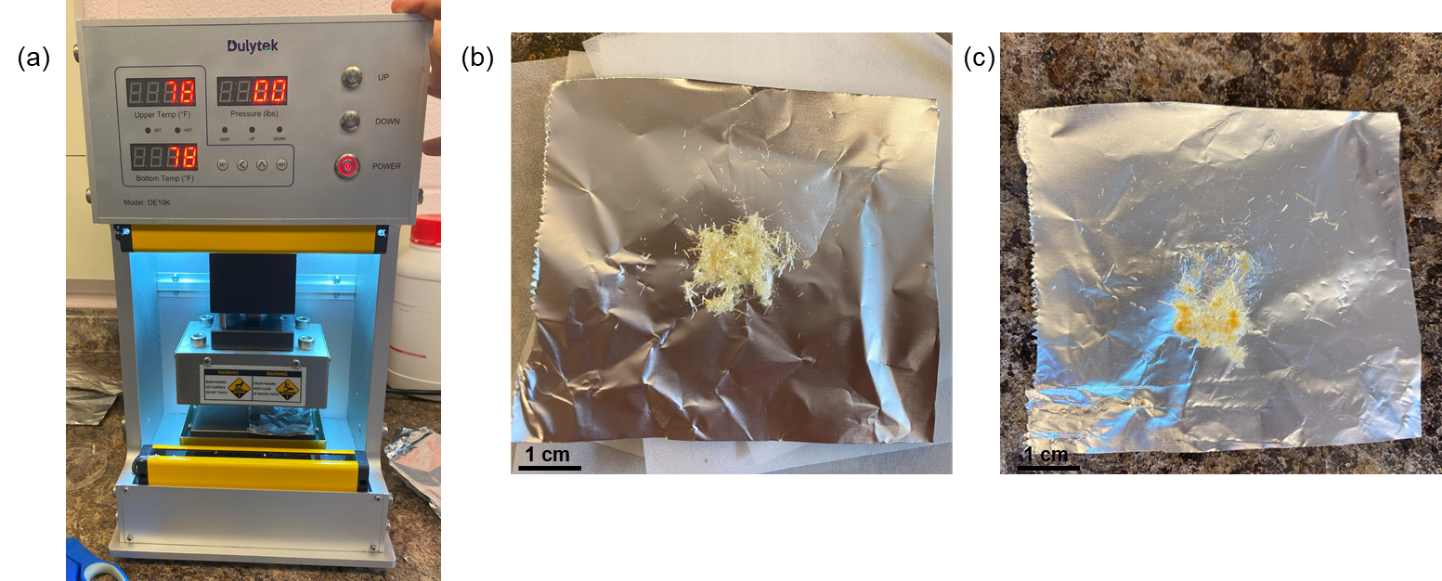


**Figure S10.** Mechanical depolymerization of PBIT polymer crystals under compression. (**a**) Hard pressing setup with Dulytek® Elite DE10K Hybrid Rosin Heat Press, 5 Tons. (**b**) PBIT-8 polymer before hard pressing. (**c**) PBIT-8 polymer after hard pressing.


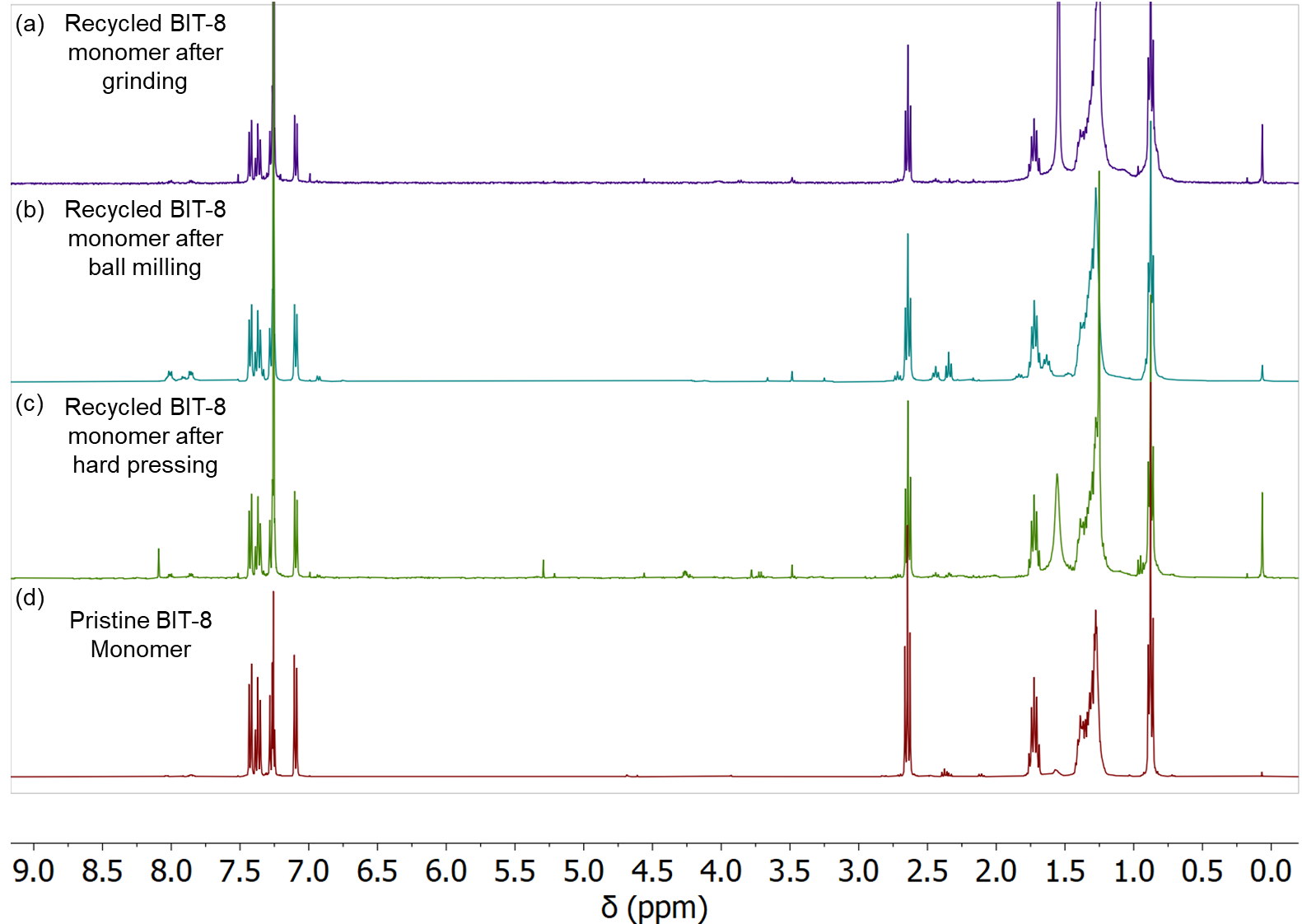


**Figure S11**. Overlays of ^1^H NMR spectra of recycled BIT-8 monomers after grinding (**a**), after ball milling (**b**), after hard pressing (**c**), and pristine monomers (**d**) (25°C, CDCl_3_, with residual solvent peaks at 7.26, 2.17, and 1.56 ppm for CHCl_3_, acetone, and H_2_O, respectively).


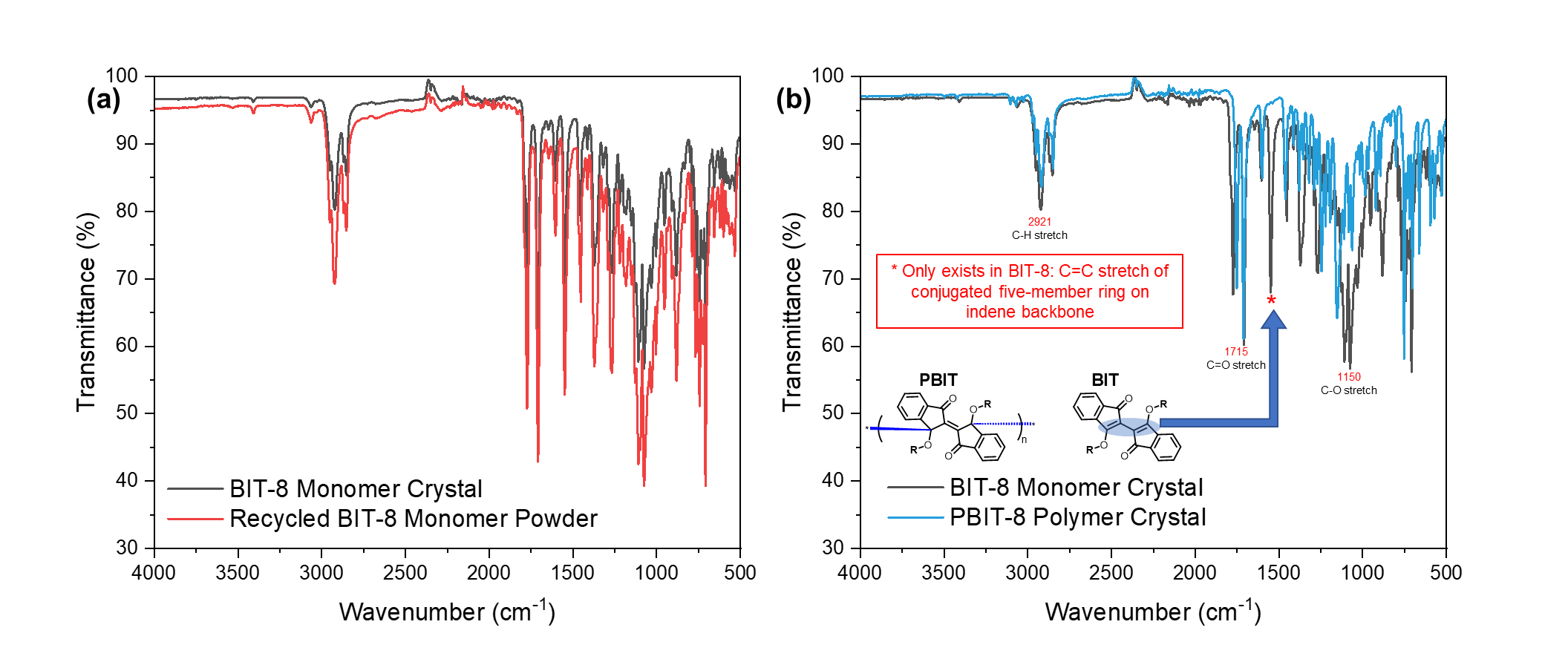


**Figure S12**. FT-IR spectra of (a) BIT-8 monomer crystal and recycled BIT-8 monomer powder after mechanical depolymerization. (b) BIT-8 monomer crystal and PBIT-8 polymer crystal.


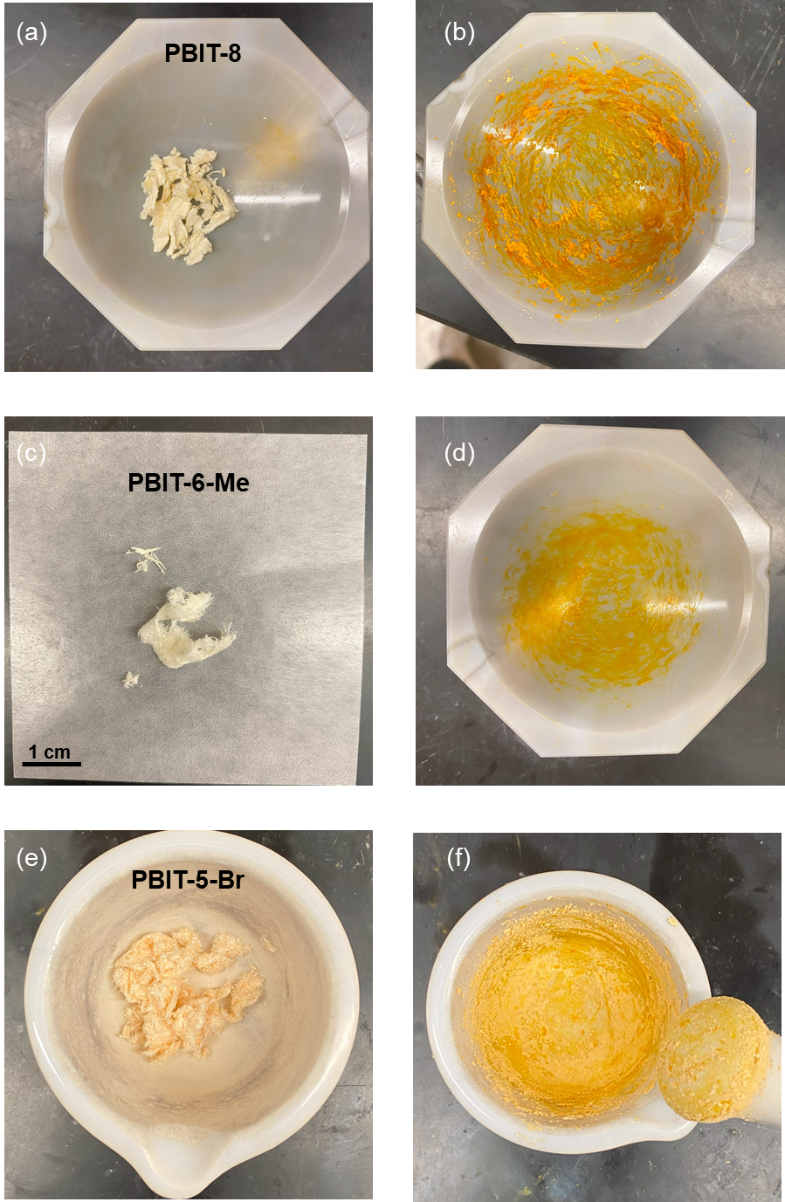


**Figure S13**. Hand grinding of various PBIT polymers. (**a**) PBIT-8 polymer before grinding. (**b**) PBIT-8 polymer after grinding for 1 minute. (**c**) PBIT-6-Me polymer before grinding. (**d**) PBIT-6-Me polymer after grinding for 1 minute. (**e**) PBIT-5-Br polymer before grinding. (**f**) PBIT-5-Br polymer after grinding for 1 minute.

*
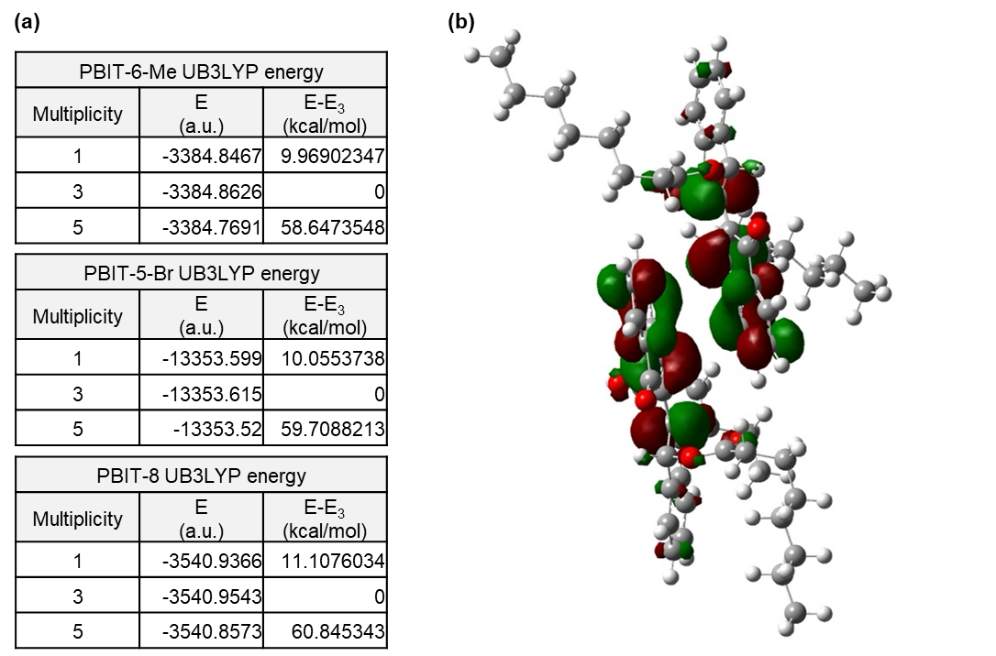
*

**Figure S14**. (a) Multiplicity test results with unrestricted method (UB3LYP) for C-C cleavage products of set A. (b). HOMO orbital distribution of BIT8 radicals (isoval = 0.02).


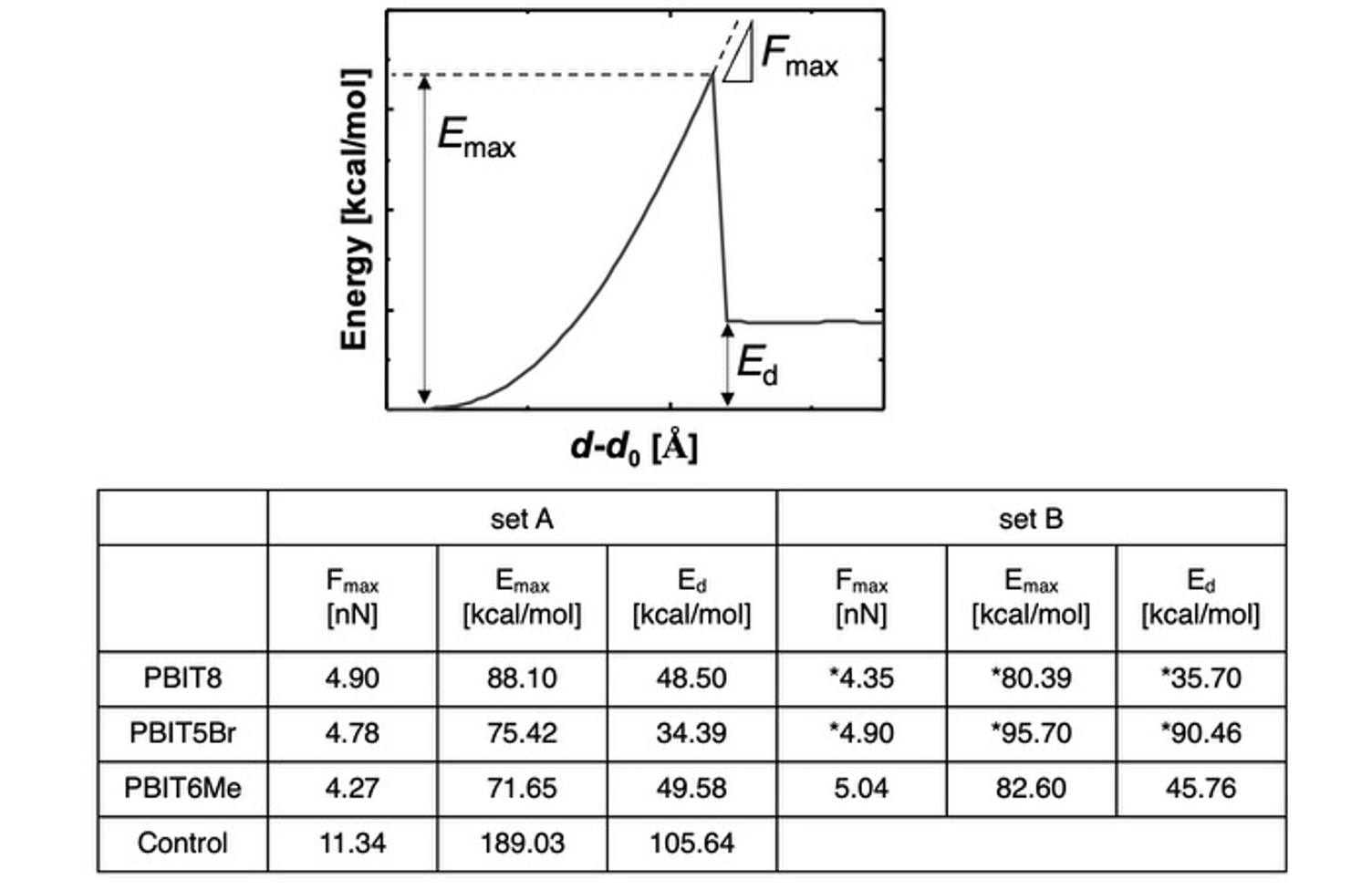


**Figure S15**. Maximum energy (Emax), force (Fmax) values, and the dissociation energy (Ed) value obtained, from CoGEF simulation (bottom). The energy-strain curve provides a graphical representation of these definitions (top). Numbers with * denotes values from reactions distinct from the C-C bond cleavage.


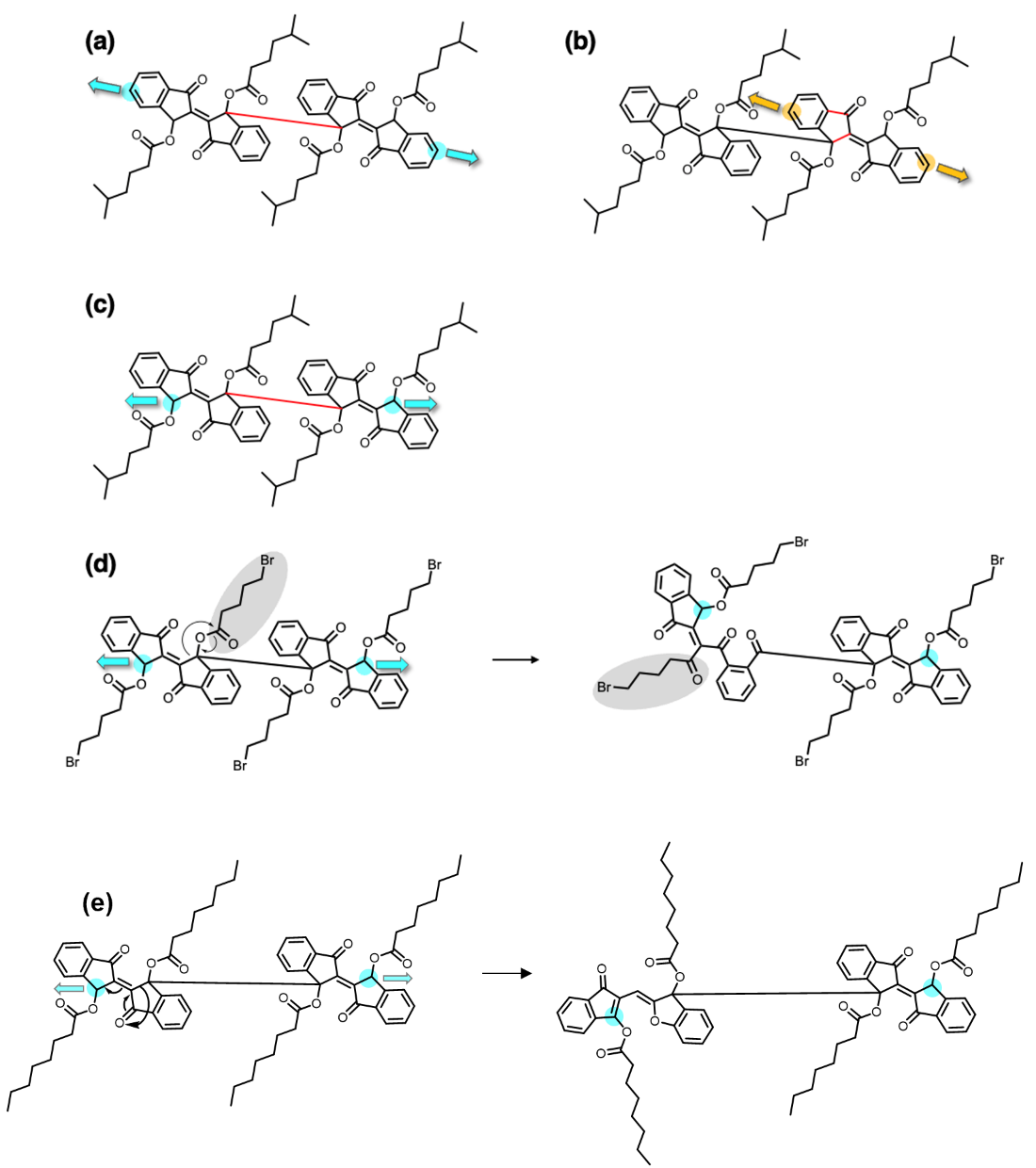


**Figure S16**. (a) Illustration of the distinct bond dissociation pattern observed between the set A stretching events within the dimer, compared to those occurring in the monomer (control simulation, b). (c-e) Illustration of the reactions happening at the first peak of pulling curves of PBIT-6-Me, PBIT-5-Br, and PBIT-8, respectively. Dissociated bonds are shown as red lines, while formed bonds are also highlighted in (d) and (e).


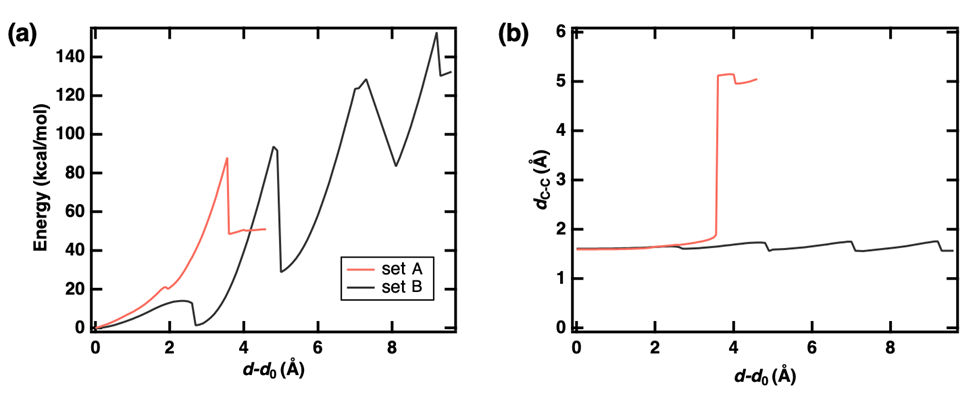


**Figure S17.** Longer observation of the set B PBIT-8 CoGEF simulation compared with the set A result. (a) Energy profile and (b) C–C bond length profile. In set B, the C–C bond does not cleave into monomer radicals, in contrast to set A.


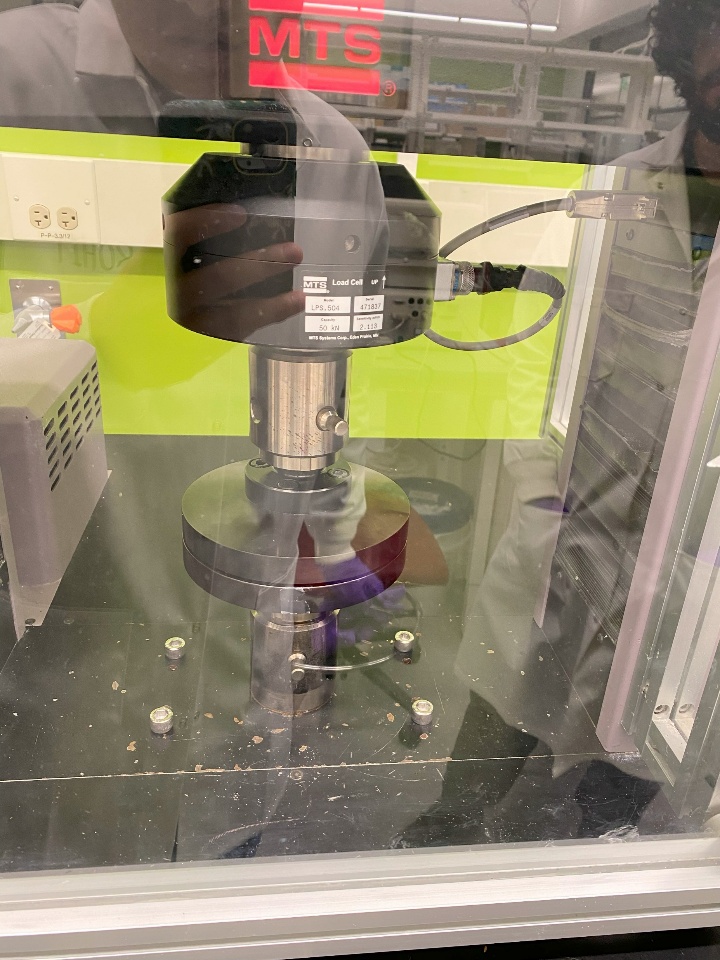


**Figure S18.** Picture of Mechanical Testing System (MTS) used for quantitative hard pressing of various PBIT thin films.


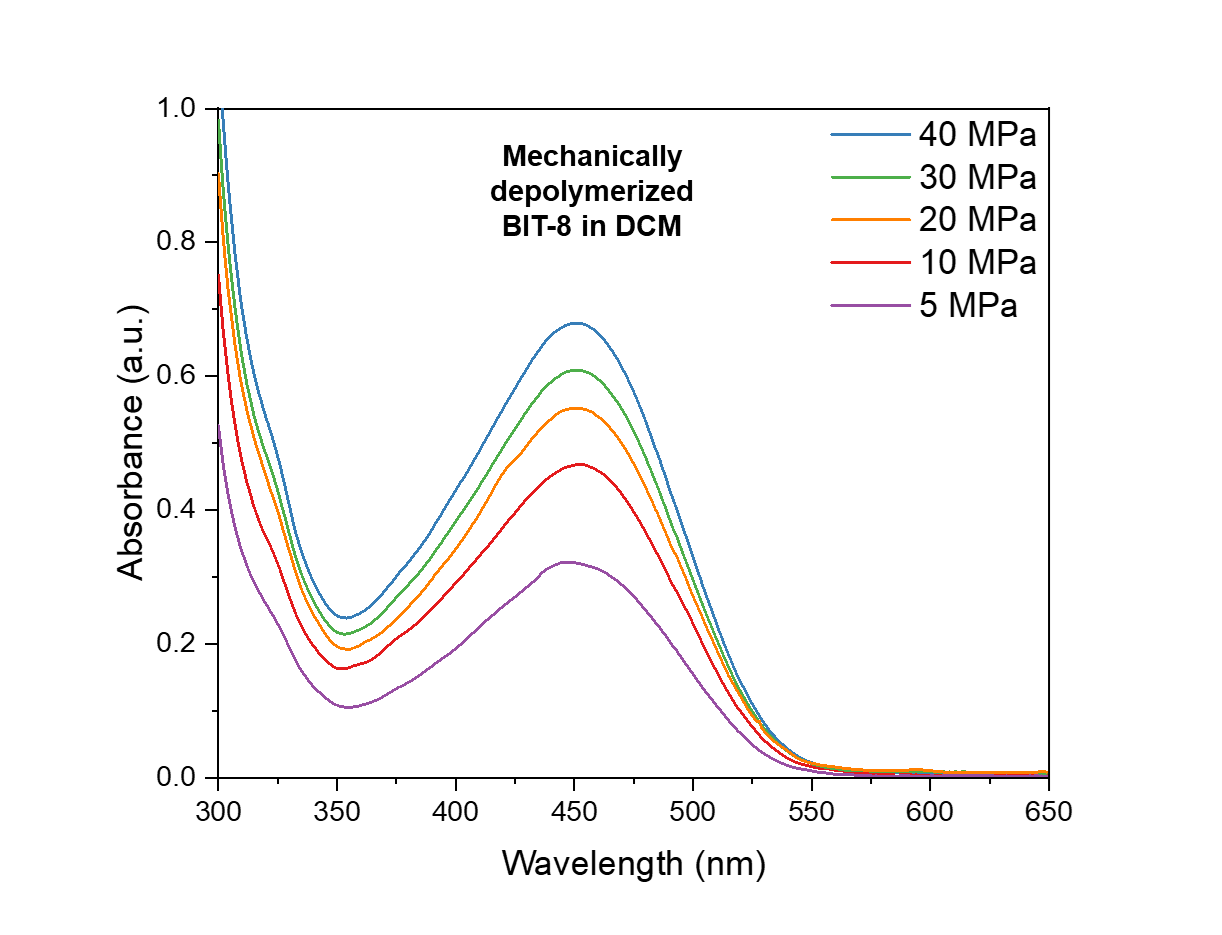


**Figure S19**. UV-Vis absorption spectra of the depolymerized BIT-8 solution in DCM under various pressures.

**
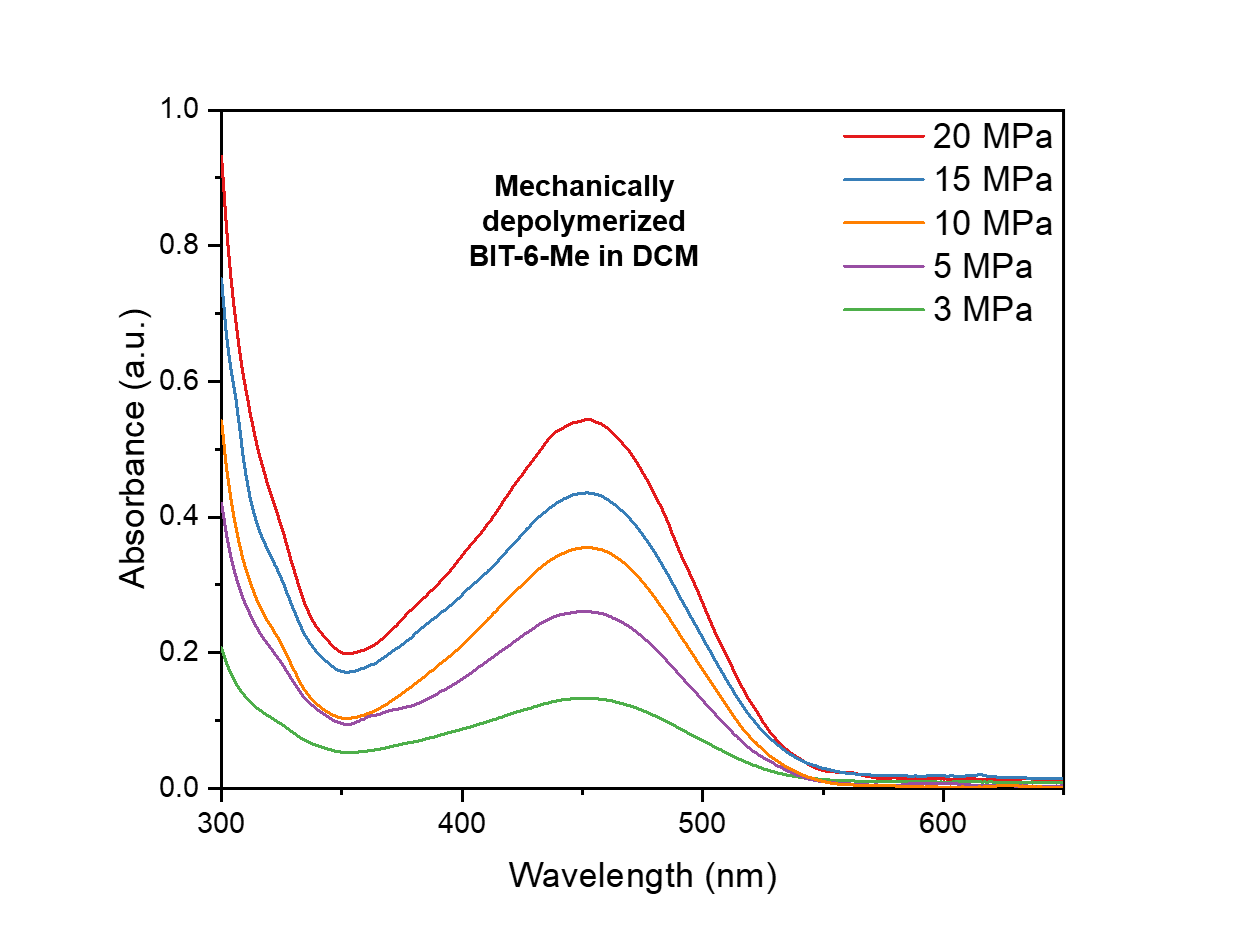
**

**Figure S20**. UV-Vis absorption spectra of the depolymerized BIT-6-Me solution in DCM under various pressures.


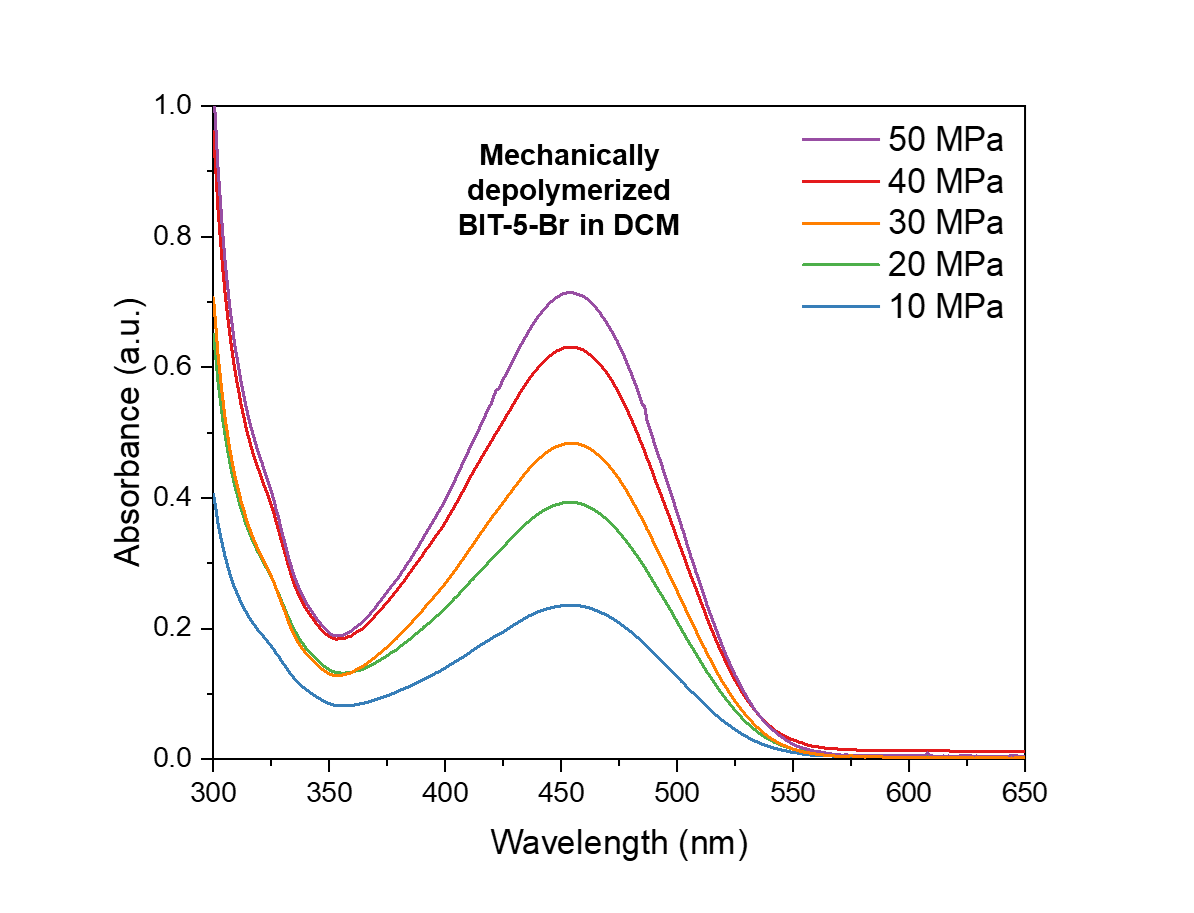


**Figure S21**. UV-Vis absorption spectra of the depolymerized BIT-5-Br solution in DCM under various pressures.


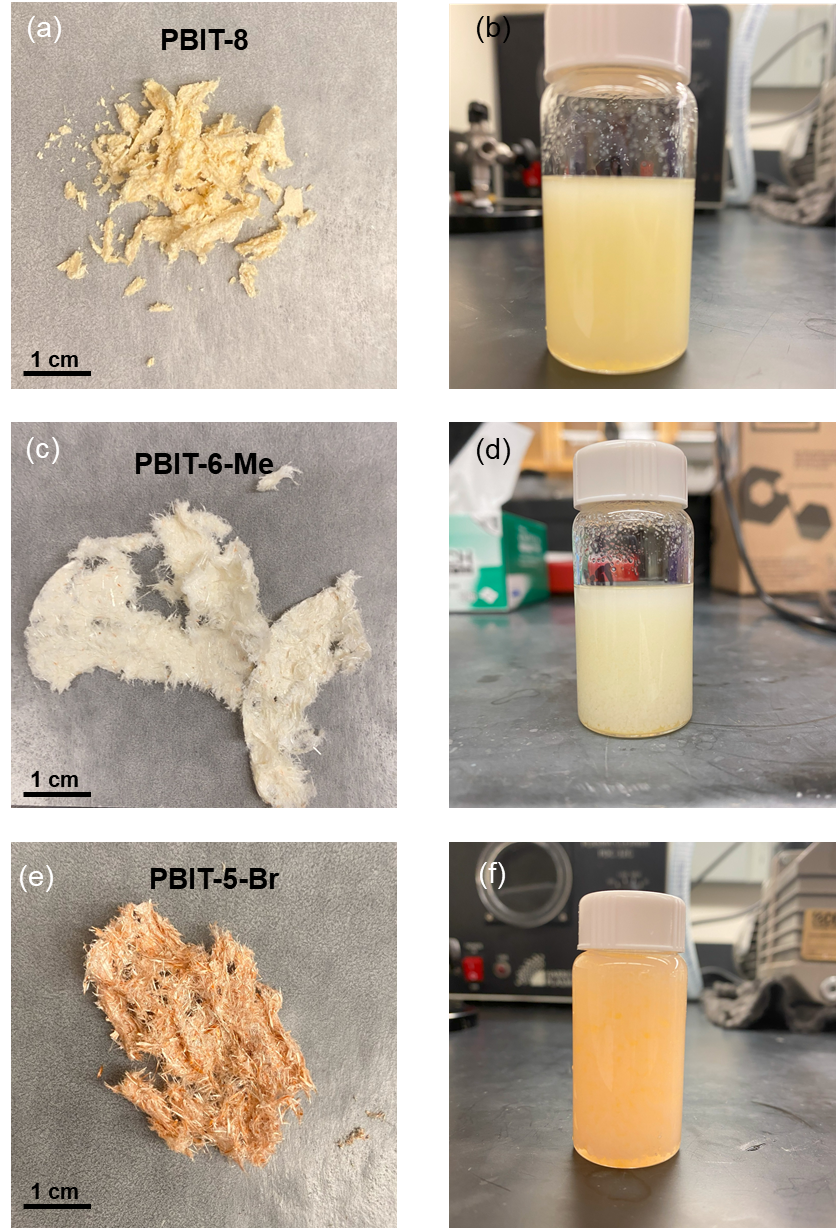


**Figure S22**. Ultrasonication of various PBIT polymers. (**a**) PBIT-8 polymer before ultrasonication. (**b**) PBIT-8 polymer after ultrasonication for 30 minutes. (**c**) PBIT-6-Me polymer before ultrasonication. (**d**) PBIT-6-Me polymer after ultrasonication for 30 minutes. (**e**) PBIT-5-Br polymer before ultrasonication. (**f**) PBIT-5-Br polymer after ultrasonication for 30 minutes.


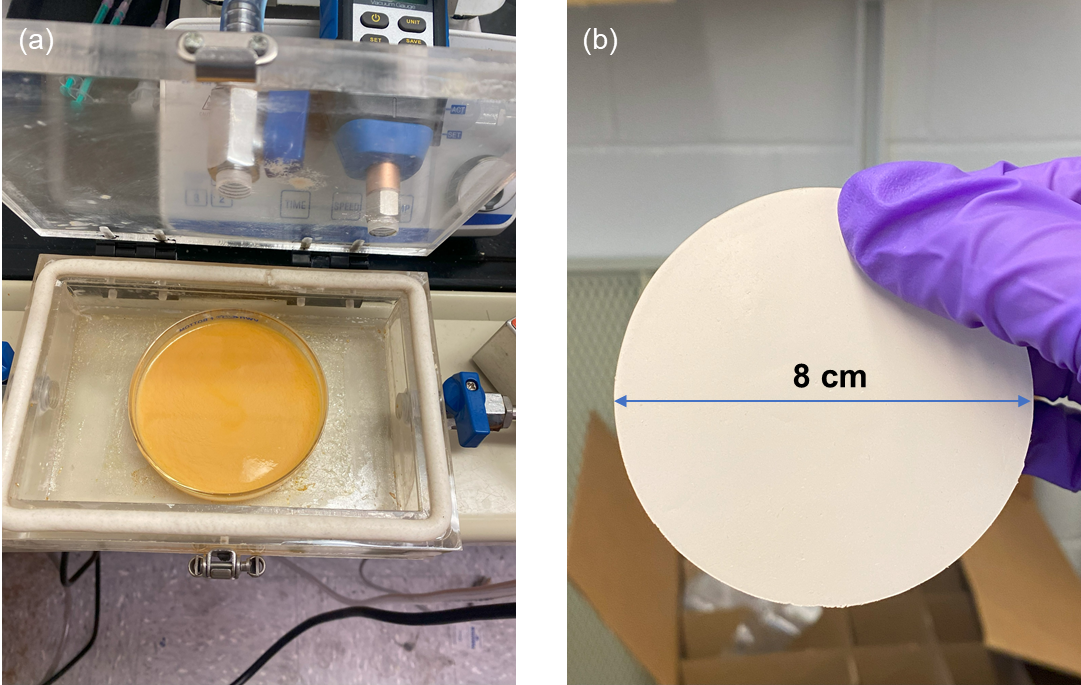


**Figure S23**. Large size PBIT-5-Br thin film fabrication processes. (**a**) PBIT-5-Br suspension after ultrasonication that were poured into a glass petri dish and put into an acrylic vacuum chamber. (**b**) PBIT-5-Br thin film after drying in the vacuum chamber and removed from the petri dish.


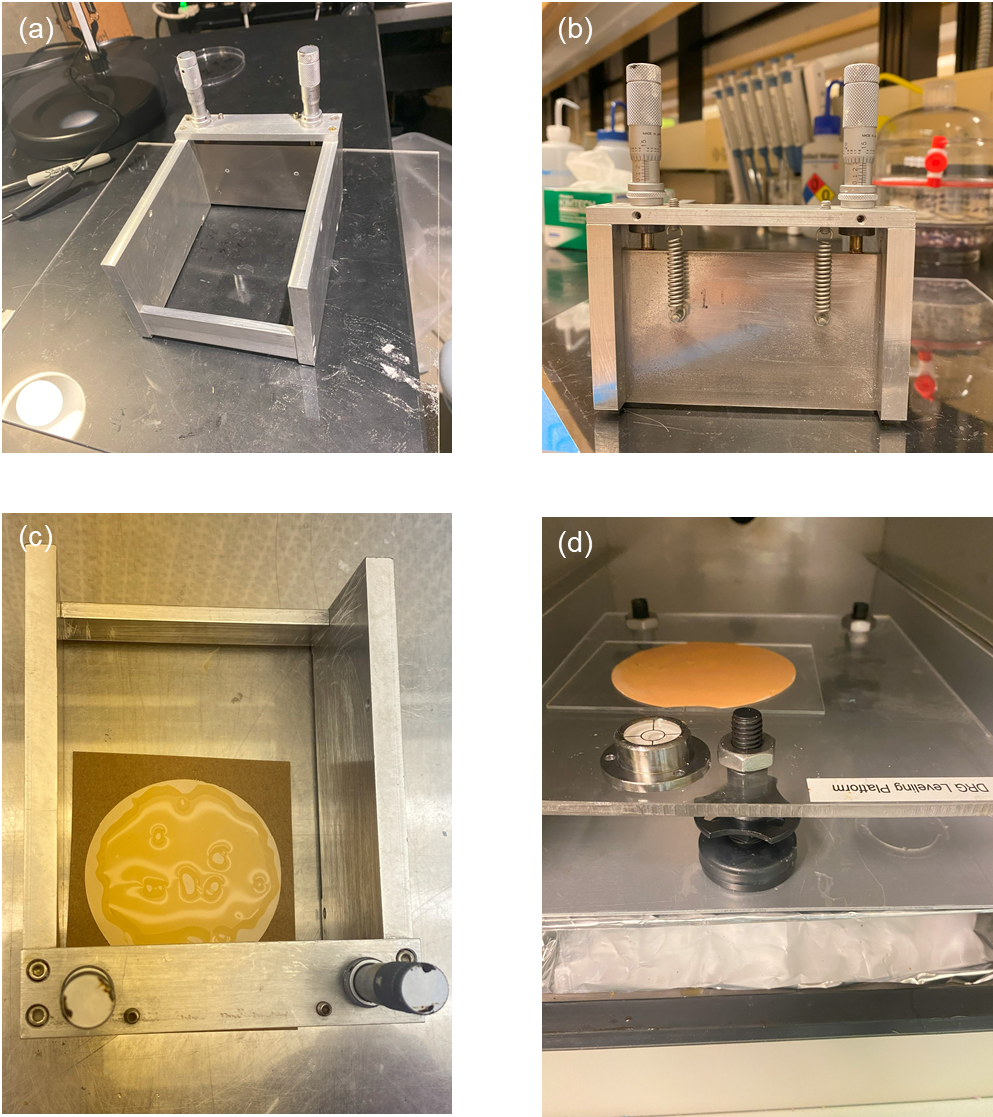


**Figure S24.** PBIT-PDMS composite film fabrications. (**a**) Top view of adjustable film applicator. (**b**) side view of adjustable film applicator. (**c**) PBIT-5-Br film in the adjustable film applicator with PDMS solution loaded on top. (**d**) PBIT-PDMS composite film during curing at 70 °C in the oven.


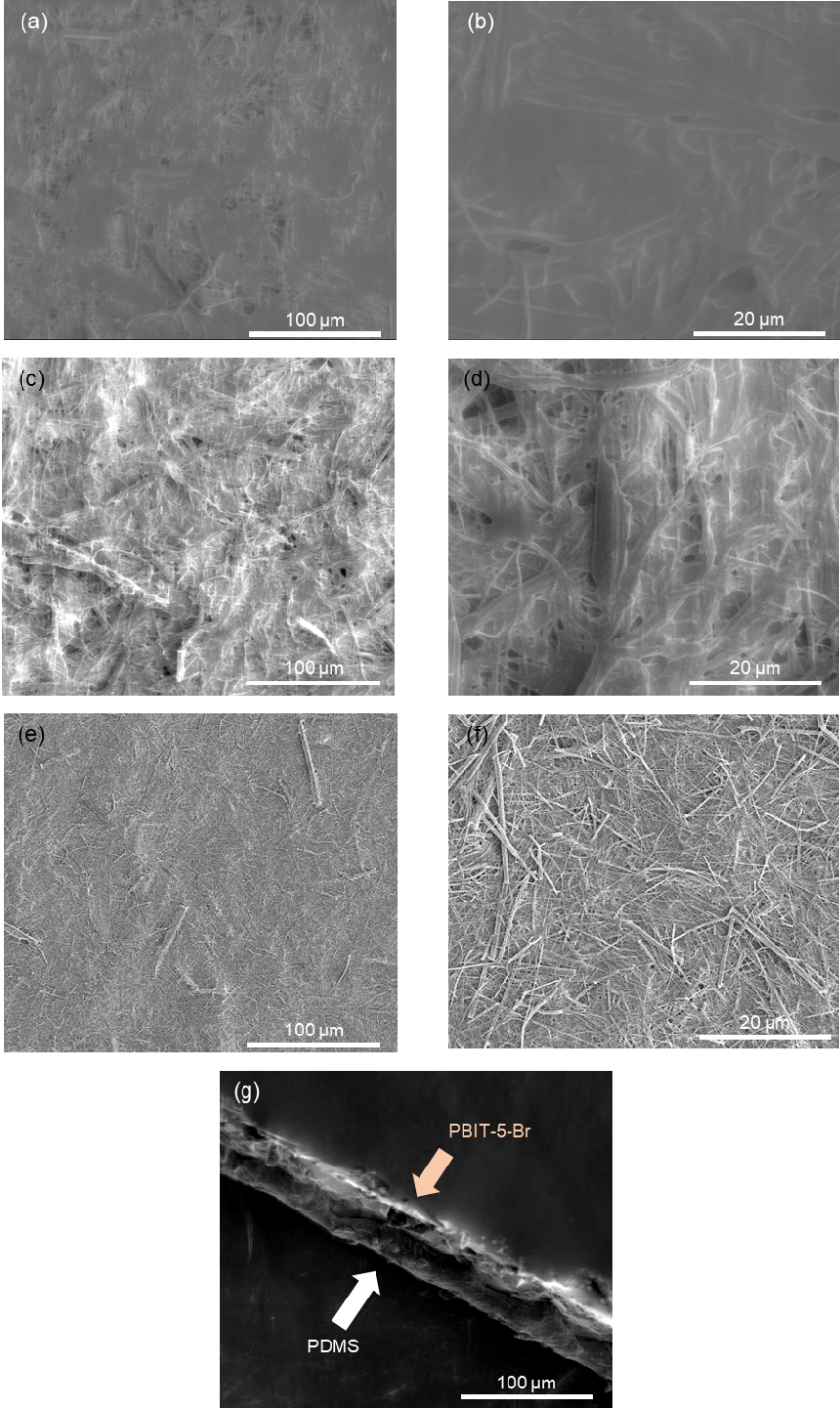


**Figure S25**. SEM images of PBIT-PDMS composite film and bare PBIT film. (**a**) Low magnification SEM image of the composite film from PDMS coated side. (**b**) High magnification SEM image of the composite film from PDMS coated side. (**c**) Low magnification SEM image of composite film from PDMS-free side. (**d**) High magnification SEM image of composite film from PDMS-free side. (**e**) Low magnification SEM image of bare PBIT-5-Br film. (**f**) High magnification SEM image of bare PBIT-5-Br film. (**g**) SEM image of cross section of the PBIT-PDMS composite film.


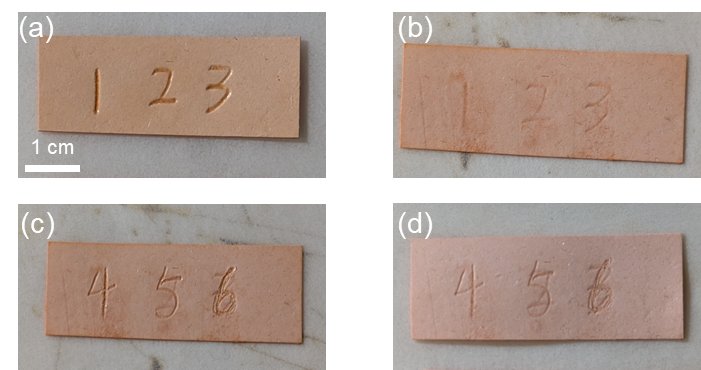


**Figure S26**. Reusability demonstration of PBIT–PDMS composite films with a PBIT layer thickness of 70 µm used as ink-free paper. (a) Composite film after first use, showing clearly visible orange writing marks. (b) Film after immersion in acetone for 5 minutes, drying, and hand pressing between cover glasses; residual traces from the initial writing remain visible. (c) Regenerated film after second round of ink-free writing, with new visible orange marks. (d) Film after second acetone wash, drying, and hand pressing; residual traces from both writing cycles remain visible.


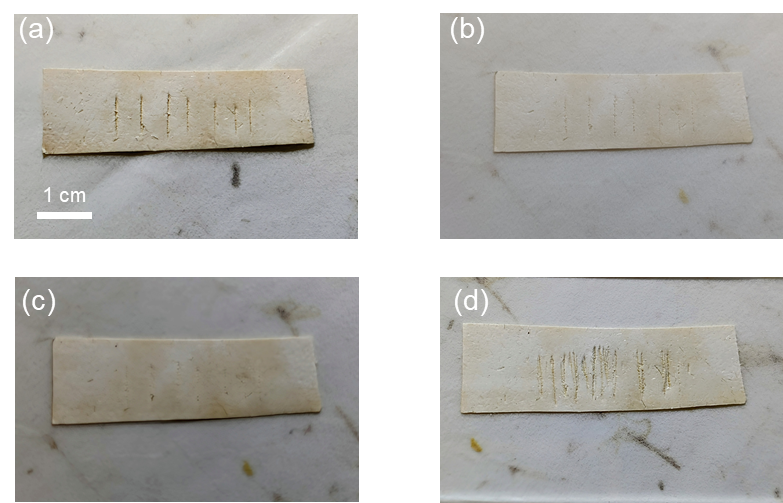


**Figure S27**. Reusability demonstration of PBIT–PDMS composite films with a PBIT layer thickness of 140 µm used as ink-free paper. (a) Composite film after first use, showing clearly visible orange writing marks. (b) Film after immersion in acetone for 5 minutes and drying; residual traces from the initial writing remain visible. (c) Washed film after hand pressing between cover glasses; residual traces are largely filled and significantly reduced. (d) Regenerated film after second round of ink-free writing, showing clear and distinct orange marks.


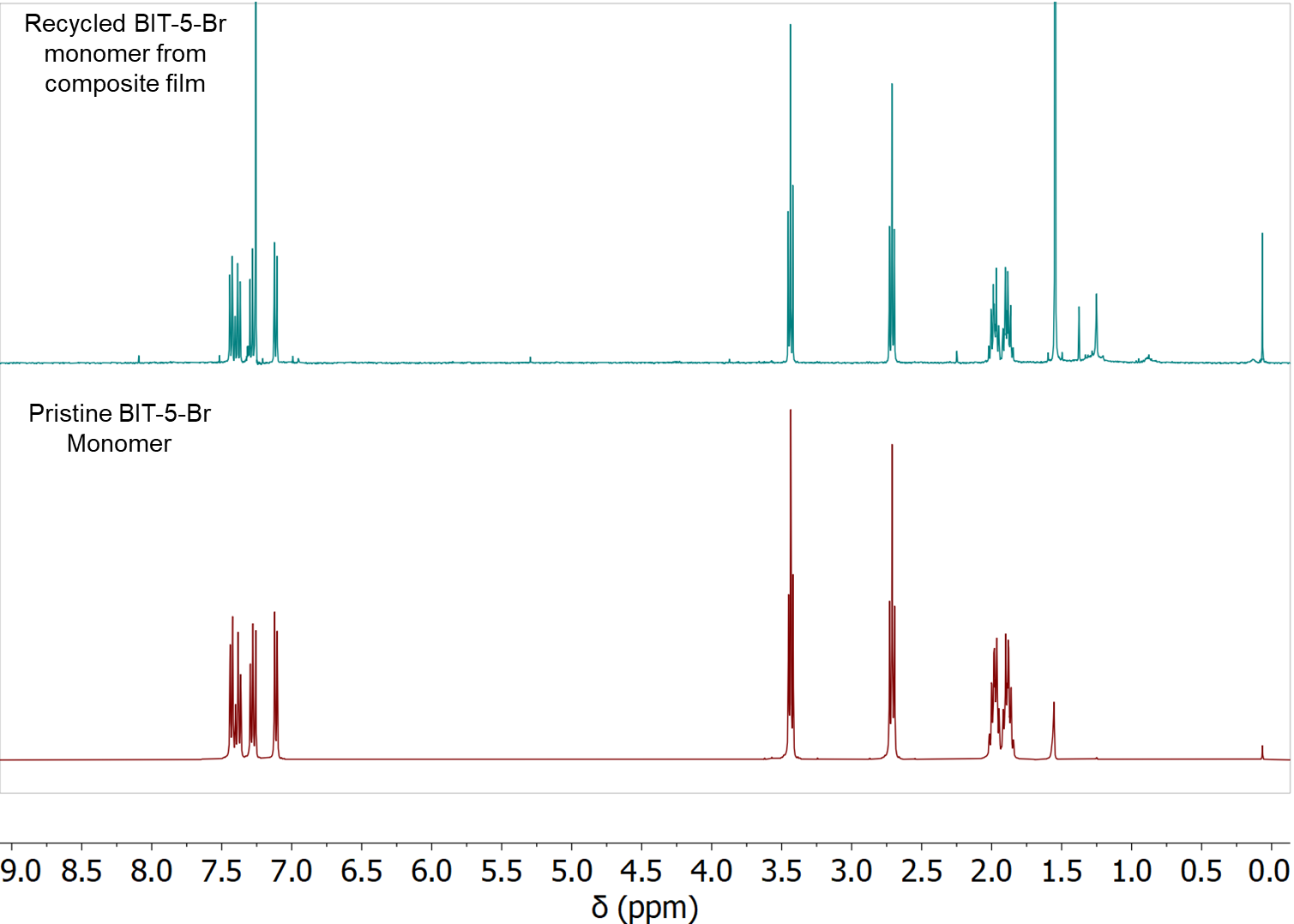


**Figure S28**. Overlays of ^1^H NMR spectra of recycled BIT-5-Br monomers from PBIT-PDMS composite film (top) and pristine monomers (bottom) (25°C, CDCl_3_, with residual solvent peaks at 7.26, 2.17, and 1.56 ppm for CHCl_3_, acetone, and H_2_O, respectively).

**References:**

(1) Dou, L.; Zheng, Y.; Shen, X.; Wu, G.; Fields, K.; Hsu, W.-C.; Zhou, H.; Yang, Y.; Wudl, F. Single-Crystal Linear Polymers Through Visible Light–Triggered Topochemical Quantitative Polymerization. *Science* **2014**, *343*, 272-277.

(2) Luo, X.; Wei, Z.; Seo, B.; Hu, Q.; Wang, X.; Romo, J. A.; Jain, M.; Cakmak, M.; Boudouris, B. W.; Zhao, K.; et al. Circularly Recyclable Polymers Featuring Topochemically Weakened Carbon–Carbon Bonds. *J. Am. Chem. Soc.* **2022**, *144*, 16588-16597.

(3) Wei, Z.; Wang, X.; Seo, B.; Luo, X.; Hu, Q.; Jones, J.; Zeller, M.; Wang, K.; Savoie, B. M.; Zhao, K.; Dou, L. Side-Chain Control of Topochemical Polymer Single Crystals with Tunable Elastic Modulus. *Angew. Chem., Int. Ed.* **2022**, *61*, e202213840.

(4) Beyer, M. K. The mechanical strength of a covalent bond calculated by density functional theory. *J. Chem. Phys.* **2000**, *112*, 7307-7312.

(5) M. J. Frisch, G. W. T., H. B. Schlegel, G. E. Scuseria, M. a. Robb, J. R. Cheeseman, G. Scalmani, V. Barone, G. a. Petersson, H. Nakatsuji, X. Li, M. Caricato, a. V. Marenich, J. Bloino, B. G. Janesko, R. Gomperts, B. Mennucci, H. P. Hratchian, J. V. Ortiz, a. F. Izmaylov, J. L. Sonnenberg, Williams, F. Ding, F. Lipparini, F. Egidi, J. Goings, B. Peng, A. Petrone, T. Henderson, D. Ranasinghe, V. G. Zakrzewski, J. Gao, N. Rega, G. Zheng, W. Liang, M. Hada, M. Ehara, K. Toyota, R. Fukuda, J. Hasegawa, M. Ishida, T. Nakajima, Y. Honda, O. Kitao, H. Nakai, T. Vreven, K. Throssell, J. a. Montgomery Jr., J. E. Peralta, F. Ogliaro, M. J. Bearpark, J. J. Heyd, E. N. Brothers, K. N. Kudin, V. N. Staroverov, T. a. Keith, R. Kobayashi, J. Normand, K. Raghavachari, a. P. Rendell, J. C. Burant, S. S. Iyengar, J. Tomasi, M. Cossi, J. M. Millam, M. Klene, C. Adamo, R. Cammi, J. W. Ochterski, R. L. Martin, K. Morokuma, O. Farkas, J. B. Foresman, D. J. Fox. Gaussian 16. **2016**,

(6) Grimme, S.; Ehrlich, S.; Goerigk, L. Effect of the damping function in dispersion corrected density functional theory. *J. Comput. Chem.* **2011**, *32*, 1456-1465.

(7) Pracht, P.; Bohle, F.; Grimme, S. Automated exploration of the low-energy chemical space with fast quantum chemical methods. *Phys. Chem. Chem. Phys.* **2020**, *22*, 7169-7192.

(8) Wick, C. R.; Topraksal, E.; Smith, D. M.; Smith, A.-S. Evaluating the predictive character of the method of constrained geometries simulate external force with density functional theory. *Forces Mech.* **2022**, *9*, 100143.
